# Advances in Interface Circuits for Self-Powered Piezoelectric Energy Harvesting Systems: A Comprehensive Review

**DOI:** 10.3390/s25134029

**Published:** 2025-06-28

**Authors:** Abdallah Al Ghazi, Achour Ouslimani, Abed-Elhak Kasbari

**Affiliations:** Quartz Laboratory, Department of Electrical and Electronic Engineering, Ecole Nationale Supérieure de l’Electronique et de ses Applications, Ensea, 6 Avenue du Ponceau, 95014 Cergy, France

**Keywords:** piezoelectric, energy harvesting, MPPT, smart rectifier, SSH, self-powered

## Abstract

This paper presents a comprehensive summary of recent advances in circuit topologies for piezoelectric energy harvesting, leading to self-powered systems (SPSs), covering the full-bridge rectifier (FBR) and half-bridge rectifier (HBR), AC-DC converters, and maximum power point tracking (MPPT) techniques. These approaches are analyzed with respect to their advantages, limitations, and overall impact on energy harvesting efficiency. Th work explores alternative methods that leverage phase shifting between voltage and current waveform components to enhance conversion performance. Additionally, it provides detailed insights into advanced design strategies, including adaptive power management algorithms, low-power control techniques, and complex impedance matching. The paper also addresses the fundamental principles and challenges of converting mechanical vibrations into electrical energy. Experimental results and performance metrics are reviewed, particularly in relation to hybrid approaches, load impedance, vibration frequency, and power conditioning requirements in energy harvesting systems. This review aims to provide researchers and engineers with a critical understanding of the current state of the art, key challenges, and emerging opportunities in piezoelectric energy harvesting. By examining recent developments, it offers valuable insights into optimizing interface circuit design for the development of efficient and self-sustaining piezoelectric energy harvesting systems.

## 1. Introduction

The field of piezoelectric energy harvesting has witnessed a surge in the development of interface circuits tailored to its unique requirements [1,2]. This area of research has garnered considerable interest thanks to the remarkable attributes it offers, including high power density, a wide voltage range, and seamless integration with IC technology. What sets these interface circuits apart is the striking contrast between the operational speeds of ICs and the rhythmic nature of mechanical vibrations. This incongruity presents an exceptional opportunity to incorporate nonlinear switching and control mechanisms into the interface circuits, thereby enhancing the extraction of power to unprecedented levels. By capitalizing on this disparity, researchers have been able to unlock the true potential of piezoelectric energy harvesting. In a piezoelectric energy-harvesting system, the input power processing circuit plays a vital role in optimizing harvested energy. Comprising the interface and harvester control, this circuit ensures compatibility with the energy storage system. Due to the AC nature of piezoelectric vibrations, an AC-to-DC rectifier is used, posing challenges in efficient power extraction. Designing rectifier circuits that effectively extract power while minimizing losses and optimizing efficiency is a task for researchers and engineers. The harvester control component enables the interface to adapt and optimize performance based on varying vibration characteristics, enhancing power extraction efficiency. By fine-tuning the interface circuit, maximum utilization of available energy can be achieved.

**Figure 1:**
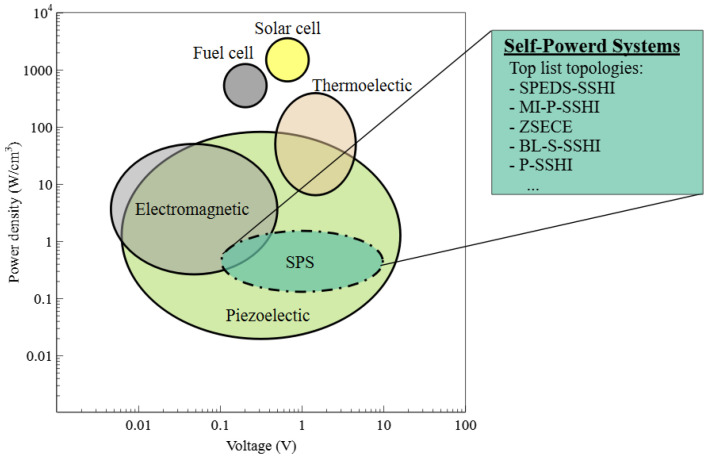
Share of each source in energy productions and its power density. The top list of SPS topologies.

This section establishes the growing importance of piezoelectric energy harvesting and outlines the primary challenges in designing effective interface circuits by emphasizing the need for innovative circuit topologies that can reconcile the mismatch between mechanical vibrations and electronic processing speeds. It also highlights the potential benefits of a comparison that includes other energy harvesting techniques—such as solar, thermoelectric, or electromagnetic methods—to further contextualize the review’s scope and objectives as depicted in Figure 1.

Researchers have made significant advancements in enhancing power extraction efficiency in piezoelectric energy harvesting systems by exploring nonlinear switching techniques. Two notable methods, synchronous electric charge extraction (SECE) and parallel synchronized switch harvesting on inductor (P-SSHI), have demonstrated promising results [1,3,4,5]. These techniques exploit synchronous switching at peak vibration displacement to unlock a substantial increase in extracted power. By introducing strategic timing delays in the switching process [6,7,8], researchers have achieved complex impedance matching, enabling improved performance even at off-resonance frequencies. This innovation expands the operational range of the energy-harvesting system, ensuring efficient power extraction across a wider spectrum of vibration frequencies.

Furthermore, the development of interfaces incorporating multiple transducers has contributed to further advancements in power extraction [9,10]. By harnessing the collective energy from multiple transducers, these interfaces significantly enhance overall power output. This approach proves particularly beneficial in scenarios where a single transducer may not generate sufficient power independently. The combination of multiple transducers with sophisticated interface designs maximizes power extraction efficiency and facilitates practical implementation of piezoelectric energy harvesting systems. The integration of nonlinear switching techniques, timing delays, complex impedance matching, and the utilization of multiple transducers opens up new possibilities for enhancing power extraction efficiency in piezoelectric energy harvesting systems, leading to independent SPSs. These innovations pave the way for more efficient and practical utilization of piezoelectric energy in various applications. Optimizing interface circuits in piezoelectric energy harvesting systems requires careful parameter adjustments, regardless of the circuit architecture. The extracted power is influenced by the effective loading on the transducer. To address this, many designs incorporate a switching power converter after the rectifier, enabling adjustment of the effective loading through modifications in the converter’s switching timing [11]. Start-up and runtime calibrations are crucial to maintain optimal performance in varying operating conditions.

Several maximum power point tracking (MPPT) algorithms have been proposed, including Perturb and Observe (P&O) and fractional $V_{OC}$, each with tradeoffs in circuit complexity, tracking accuracy, convergence time, and power consumption [12,13]. This article introduces cutting-edge interface designs and MPPT methods for piezoelectric energy harvesting, emphasizing important considerations in circuit implementation. It provides valuable insights for optimizing circuit performance and achieving efficient power extraction from piezoelectric systems.

This paper systematically investigates the latest advancements in piezoelectric energy harvesting (PEH) systems and their interface circuits, addressing the inherent challenges of transforming mechanical vibrations into usable electrical energy by conducting a comprehensive literature review. To achieve this, a systematic analysis of interface circuits for self-powered piezoelectric energy harvesting was performed, employing comprehensive searches across academic databases including IEEE Xplore, MDPI, Scopus, Web of Science, and Google Scholar. This search strategy utilized a targeted approach, employing keywords and combinations such as ‘’piezoelectric energy harvesting”, ‘’self-powered systems”, ‘’interface circuit”, ‘’power management”, ‘’rectifier”, ‘’dc–dc converter”, ‘’MPPT”, ‘’SECE”, and ‘’SSH”. The review included peer-reviewed journal articles and conference proceedings focusing on interface circuit design, experimental validation, and self-powered system applications published between 2021 and 2025, ensuring the capture of the most relevant and recent contributions, while excluding purely theoretical studies or those solely focused on materials and fabrication without circuit analysis.

The article is organized as follows:

Section 2 lays out the fundamental principles of PEH and introduces the equivalent circuit model of a piezoelectric transducer. It discusses the challenges associated with AC-DC conversion, energy storage, voltage regulation, impedance matching, and the influence of load dependency on power extraction efficiency. Section 3 examines basic interface circuits like full-bridge rectifiers (FBRs) and half-bridge rectifiers (HBRs), analyzing their advantages, limitations, and impact on energy harvesting efficiency. Section 4 delves into advanced interface circuits, specifically Synchronous Switch Harvesting (SSH) techniques, which employ “switching manipulation” to optimize power extraction. It explores both open-circuit and short-circuit SSH interfaces, including Synchronous Electric Charge Extraction (SECE), predamping techniques (like S-SSHI), energy investing/pileup methods, and parallel SSHI (P-SSHI). The section discusses the principles, circuit topologies, control schemes, and performance improvements of these techniques. It also examines Maximum Power Point Tracking (MPPT) methods, such as fractional VOC and Perturb and Observe (P&O), for maximizing power extraction under varying load conditions. Additionally, it explores techniques for nonresonant operation, addressing challenges when the operating frequency deviates from the piezo structure’s resonant frequency.

Section 5 examines self-powered systems (SPSs) for PEH, categorizing them into single and array systems. For single SPSs, it discusses circuit implementations, efficiency, and power levels. For array SPSs, it looks into systems with multiple inputs, including thebenefits and challenges of using multiple transducers with different orientations, resonantfrequencies, and electrode configurations.

Section 6 provides a comparative analysis of the various interface circuits discussed in the previous sections, highlighting key features, advantages, and limitations. It discusses the importance of considering factors such as the number of switches, breakdown voltage limitations, calibration methods, power consumption, frequency deviations, and multi-input capabilities when selecting and designing interface circuits for specific PEH applications. The article concludes with a discussion of future research directions and the need for a holistic approach to optimize PEH systems, considering factors like environmental impact, power consumption, and real-world validation.

The article concludes with Section 7.

## 2. Equivalent Circuit Model

**Figure 2:**
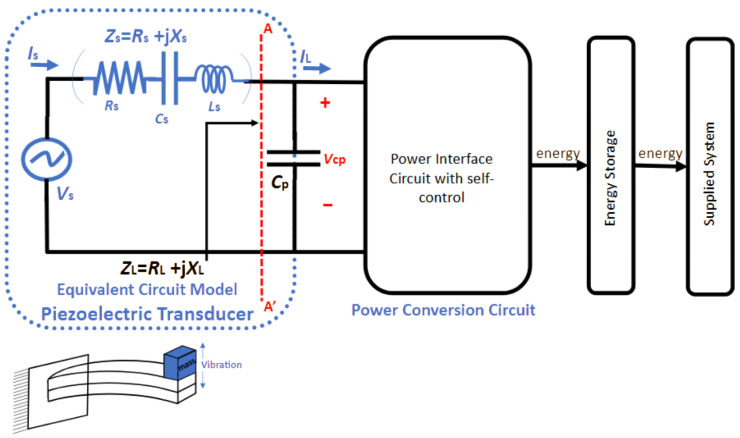
Configuration of a piezoelectric transducer: cantilever beam with mounting mass and equivalent circuit model with power processing circuit.

Piezoelectric materials have the remarkable ability to convert mechanical strain into electrical charges [14]. One commonly used configuration for piezoelectric transducers is a cantilever beam with a mounted mass (Figure 2). Shocks or vibrations applied to the system cause the attached mass to oscillate, generating strain in the piezoelectric material. By connecting the electrodes of the piezoelectric cantilever to a load through conductive wires, the generated charges form an electric current loop, enabling the transmission of power to the connected system. To gain a deeper understanding of this system’s behavior, a mathematical model is employed, considering the distinct densities of its components and assuming vibrations occur near the system’s resonant frequency. This model includes a mass, spring, damper, and the piezoelectric system itself [15]. The governing equations of this model establish the interrelationships between parameters, providing a powerful tool for analyzing and predicting the system’s behavior. This mathematical framework empowers researchers and engineers to comprehend the underlying dynamics and optimize the performance of piezoelectric energy harvesting systems.

(1) $$Mu^{\prime \prime }\left(t\right)+\nu u^{\prime }\left(t\right)+Ku\left(t\right)+\theta V_{CP}\left(t\right)=F\left(t\right)$$

(2) $$-\theta u^{\prime }\left(t\right)+C_{P}V_{CP}^{\prime }\left(t\right)=-I_{L}\left(t\right)$$

The piezoelectric transducer can be effectively represented by an equivalent circuit model using Equations (1) and (2), as illustrated in Figure 2. The equation involves several parameters: mass (M), mechanical damping (ν), stiffness (K), piezoelectric coefficient (θ), and capacitance ($C_{P}$). The external force applied to the system is represented by F(t). This force can be a constant vibration (sinusoidal) or a sudden shock (impulsive). The system’s response includes the mass displacement (u(t)), voltage across the piezoelectric electrodes ($V_{CP}\left(t\right)$), and current flowing through them ($I_{L}\left(t\right)$). This model incorporates parameters such as $R_{S}$ = $\nu /\theta^{2}$, $L_{s}$ = $M/\theta^{2}$, $C_{s}$ = θ/K, and $C_{P}$, which represent the equivalent resistance, inductance, and capacitances, respectively. The input voltage is denoted as $V_{S}$ = $F_{0}/\theta$, and the electromechanical interface is depicted by the red line *A*-$A^{\prime }$. The transducer structure exhibits two resonant frequencies: the open-circuit resonant frequency $\left(\omega_{OC}=1/\sqrt{\frac{L_{s}C_{s}C_{P}}{C_{s}+C_{P}}}\right)$ and the short-circuit resonant frequency ($\omega_{SC}=1/\sqrt{L_{s}C_{s}}$). These frequencies provide important insights into the resonant behavior of the transducer and play a crucial role in its performance analysis and optimization.

The equivalent circuit model section provides a detailed framework for understanding the piezoelectric transducer’s behavior. Key parameters—including mass, damping, stiffness, piezoelectric coefficient, and capacitance—are defined and related through governing equations. This summary underscores how these elements combine to form a model that is critical for predicting system performance and optimizing energy conversion.

The power processing circuit, depicted in Figure 2, plays a crucial role in conditioning the extracted power for transfer to the connected system. This circuit performs AC-DC conversion, energy storage, and voltage regulation. It also determines the load seen by the transducer. According to the maximum power transfer (MPT) theorem, the power processing circuit, represented by impedance $Z_{L}=R_{L}+jX_{L}$, maximizes power transfer when it is a complex conjugate of the impedance on the other side of the electromechanical interface (*A*-$A^{\prime }$). This condition, expressed as $Z_{L}=Z_{S}^{*}={\left(R_{S}+jX_{S}\right)}^{*}$ [14], ensures efficient power transfer. At maximum power transfer, the extracted power ideally matches the theoretical available power ($P_{AVL}$) given by $V_{S}^{2}/8R_{S}$ = $F_{0}^{2}/8\nu$, where $F_{0}$ is the force and ν is the mechanical damping coefficient. However, in practical scenarios, the actual extracted power and electrical behavior are significantly influenced by the operations of the interface.

In recent years, significant progress has been made in enhancing the performance of interface circuits, mirroring the trends highlighted previously. Following the trends discussed in this article, which encompass various aspects of interface circuit enhancement, recent developments showcase advancements across several innovative circuit topologies. For example, the SPEDS-SSHI circuit in Qi et al. [16] exemplifies progress in simultaneous energy harvesting from multiple PZTs and self-powered operation, achieving improved efficiency through component reuse, although practical validation requires further attention. Similarly, the Multi-input P-SSHI interface circuit in Wang et al. [17] presents an efficient and simplified rectifier topology with self-powering capabilities, yet its scalability and robustness are areas for future investigation. Conversely, the Self-powered Extensible SECE Rectifier in Qiu et al. [18] demonstrates load-independent power output and enhanced energy extraction, albeit with increased complexity and implementation costs. Furthermore, designs like the Self-powered Multi-Input Bridgeless series SSHI Circuit in Wu et al. [19], leveraging voltage doubler topologies, achieve notable power increases but often lack comprehensive experimental details and face scalability challenges. Even optimized topologies such as the Self-Powered P-SSHI Circuit in Zouari et al. [20], which enhance efficiency via transistor replacements, still present methodological gaps and validation needs. These diverse advancements, alongside emerging intelligent control strategies like AI-powered MPPT algorithms, underscore the continuous evolution toward more sophisticated interface circuit technology. However, these developments also emphasize the ongoing need for future research to address not only performance enhancement but also crucial practical considerations such as scalability, robust control methodologies, comprehensive experimental validation, and real-world applicability.

## 3. Basic Interface Circuits

### 3.1. Full-Bridge Rectifiers

**Figure 3:**
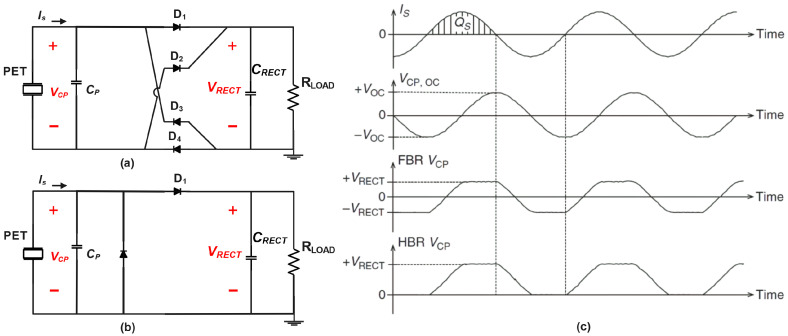
AC-to-DC voltage conversion: (**a**) Full-bridge and (**b**) half-bridge rectifier circuits, (**c**) Waveforms of the interface current ($I_{S}$) and the voltage across the piezoelectric electrodes ($V_{CP}$) with and without a rectifier.

To convert the alternating current (AC) generated by a piezoelectric transducer into a direct current (DC) voltage suitable for application circuits, a rectifier is essential. A widely used and simple configuration is the full-bridge rectifier (FBR), as shown in Figure 3. To maintain a stable voltage, $V_{\mathrm{RECT}}$, during the quasi-steady state, a large capacitor, $C_{\mathrm{RECT}}$, is connected at the rectifier’s output. Figure 3 illustrates the waveforms of the interface current $I_{S}$, and the voltage across the piezoelectric electrodes, $V_{CP}$, both with and without the rectifier. In the equivalent model depicted in Figure 3, the interface current $I_{S}$ is influenced by the operating frequency and the equivalent load, $Z_{L}$. However, in practical situations where the real part of the load impedance $Z_{L}$ is much smaller than the real part of the source impedance (i.e., the internal impedance of the piezoelectric harvester), the system is far from impedance matching, and the extracted power is often significantly lower than the theoretically available power ($P_{\mathrm{AVL}}$). This impedance mismatch can occur when the electromechanical coupling of the harvester is weak or when the harvester is operating far from its resonant frequency. Under these conditions, where the real part of $Z_{L}$ is negligible compared to the real part of the source impedance, $I_{S}$ can be approximated as a constant and independent of the extracted power [21].

Under unloaded conditions, the transducer experiences an open-circuit load. During the positive half-cycle, $I_{S}$ charges $C_{P}$ to $+V_{OC}$, while during the negative half-cycle it discharges $C_{P}$ to $-V_{OC}$. Consequently, a peak-to-peak open-circuit voltage of $2V_{OC}$ is generated. Conversely, when a rectifier is utilized, diodes $D_{1}$ and $D_{4}$ turn on when $V_{CP}$ reaches $V_{RECT}$ (assuming zero diode voltage drop), directing $I_{S}$ to $C_{\mathrm{RECT}}$. Similarly, during the negative half-cycle of the current, $D_{2}$ and $D_{3}$ turn on when $V_{CP}$ is discharged to $-V_{RECT}$, again channeling $I_{S}$ to $C_{\mathrm{RECT}}$. As a result, only a portion of the charges are delivered to the load, while some are lost in the charging and discharging process of $C_{P}$. The harvested energy on $C_{\mathrm{RECT}}$ per half cycle can be calculated using the following expression:

(3) $$E_{\mathrm{half}-\mathrm{cycle}\;\mathrm{FB}}=\frac{C_{P}V_{OC}}{2}-2C_{P}{\left(V_{\mathrm{RECT}}-\frac{V_{OC}}{2}\right)}^{2}$$

This energy reaches its peak when $V_{\mathrm{RECT}}=V_{OC}/2$, and the maximum extracted power is $C_{P}\cdot V_{OC}\cdot f/2$, where *f* represents the frequency.

The full-bridge rectifier (FBR) is widely adopted for its simplicity and passive operation, serving as a benchmark for other interface circuits. However, it does have limitations. Firstly, it requires a Maximum Power Point Tracking (MPPT) circuit to set the rectifier voltage $V_{\mathrm{RECT}}$ at half of the open-circuit voltage $V_{\mathrm{OC}}$. Secondly, the maximum output power of the FBR depends on the capacitance $C_{P}$ (a near-constant parameter) and the open-circuit voltage $V_{\mathrm{OC}}$, the latter of which is influenced by the operating frequency due to its dependence on mechanical excitation. In practical scenarios involving piezoelectric transducers with low electromechanical coupling (e.g., typical capacitance of around 10 nF and operating frequencies in the range of 10–100 Hz), the resulting output power is often limited to less than 20% of the theoretically available power $P_{\mathrm{AVL}}$. However, for systems with high electromechanical coupling (e.g., using monocrystalline materials or optimized mechanical designs), the FBR can achieve near-maximum power output.

Additionally, the diode voltage drop ($V_{D}$), typically around 0.7 V for silicon diodes, significantly impacts the efficiency of the rectifier, particularly in low-voltage systems. For example, when $V_{\mathrm{RECT}}$ is comparable to $2V_{D}$, the charging voltage needed for $V_{CP}$ increases to $V_{\mathrm{RECT}}+2V_{D}$, reducing the extracted power substantially. While the use of active switches (e.g., in synchronous rectification) can mitigate these diode losses and improve efficiency, this approach introduces additional design complexity and requires control power, which may not be suitable for all energy-harvesting applications. Active switches, such as MOSFETs used in synchronous rectification, are particularly advantageous in scenarios where $V_{\mathrm{RECT}}$ is low and power levels are sufficient to support their overhead. However, in many practical applications, passive diode-based rectifiers remain a simpler and more robust choice, albeit with inherent efficiency limitations.

### 3.2. Half-Bridge Rectifiers

To address diode losses without the need for active switches, an alternative solution is the half-bridge rectifier (HBR), depicted in Figure 3. With the HBR, during the positive half-cycle of the interface current ($I_{S}$), the voltage across the piezoelectric electrodes ($V_{CP}$) only needs to reach $\left(V_{\mathrm{RECT}}+V_{D}\right)$ before a single diode ($D_{1}$) turns on, directing $I_{S}$ to the capacitor $\left(C_{\mathrm{RECT}}\right)$. In the negative half-cycle, $V_{CP}$ is discharged to $-V_{D}$ when diode $D_{2}$ conducts. Notably, no charges flow to $C_{\mathrm{RECT}}$ during the negative current cycle.

The harvested energy per cycle using the HBR can be calculated using Equation (4):

(4) $$E_{\mathrm{half}-\mathrm{cycle}\;\mathrm{HB}}=C_{P}V_{OC}^{2}-C_{P}{\left(V_{\mathrm{RECT}}-V_{OC}\right)}^{2}$$

In the case of $V_{D}$ = 0, the maximum extracted power with the HBR is $\left(C_{P}\cdot V_{OC}\cdot f/2\right)$ when $\left(V_{\mathrm{RECT}}=V_{OC}\right)$. While the HBR only harvests charges during the positive current cycles, its maximum extracted power is equal to that of an FBR with twice the interface voltage. Thus, it is sometimes referred to as a “voltage doubler”. Accounting for the diode voltage drop $\left(V_{D}\right)$, the HBR delivers more power than the FBR while experiencing fewer losses and achieving higher power conversion efficiency. However, in both cases, the maximum extracted power remains significantly lower than $P_{\mathrm{AVL}}$ from the piezoelectric transducer. Subsequent sections discuss more efficient interface circuits.

In this section, the article reviews traditional rectifier approaches, namely full-bridge and half-bridge rectifiers. It summarizes their operational principles, highlighting the simplicity and inherent limitations of these circuits, such as diode voltage drops and impedance mismatches, which often restrict power extraction to below the theoretical maximum.

## 4. “Switching Key” Toward Advanced Circuits Interfaces: From SSD to SSH

Synchronous Switch Harvesting (SSH) interfaces, derived from the synchronized switch damping (SSD) technique, have gained attention for their superior power harvesting compared to traditional full-bridge rectifiers (FBRs) and half-bridge rectifiers (HBRs). The SSD technique includes two main schemes: SSD short-circuit (SSD-S) and SSD inductor (SSD-I). In SSD-S, a strategically placed switch ($S_{1}$) neutralizes accumulated electrical charges on the piezoelectric electrodes, dissipating the energy as heat in the switch resistance and reducing mechanical vibrations. These SSH interfaces effectively manage charges and minimize vibrations, offering promising potential for optimizing energy extraction from piezoelectric transducers in diverse applications.

**Figure 4:**
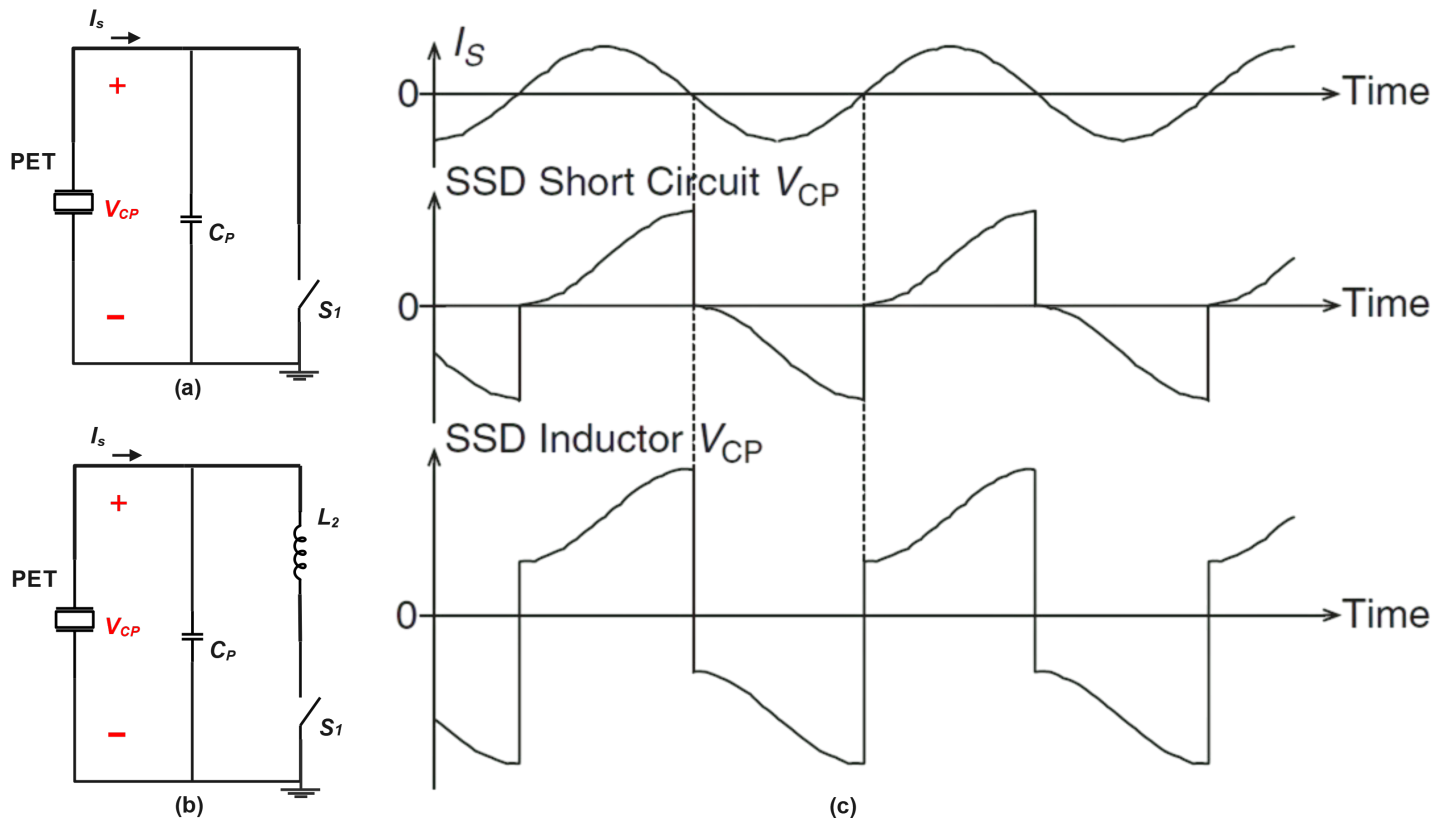
The schematics of an (**a**) SSD short circuit and (**b**) SSD inductor as well as the (**c**) associated current and voltage waveforms.

In the SSD-I Figure 4b, an inductor ($L_{2}$) is introduced in series with the switch ($S_{1}$), converting electrical potential energy to magnetic energy. This generates a piezoelectric force that counteracts the original excitation force, resulting in more effective energy attenuation and vibration reduction compared to SSD-S in Figure 4a.

Motivated by the SSD concept, SSH interfaces aim to harvest energy from mechanical vibrations while minimizing energy wastage during active switched damping. Various circuit topologies and control schemes have been proposed for SSH operations. However, precise switching and control circuits are required, and a common challenge lies in cold start-up situations when the system is deeply drained. To overcome this challenge, a parallel passive charging path is often incorporated alongside the primary power path.

SSH techniques can be categorized into two groups: open-circuit and short-circuit types. The open-circuit type, such as SECE and series SSHI, operates with the transducer under an open-circuit load most of the time. By optimizing the system to operate at the open-circuit voltage with Ω = when the series combination of inductor ($L_{s}$), capacitor ($C_{s}$) and ($C_{P}$) reaches zero impedance, the current ($I_{s}$) can be maximized.

Conversely, the short-circuit type of SSH interfaces, including parallel SSHI and SSHC, regulate the voltage across $C_{P}$ by shorting the electrodes through the rectifier. By regulating the voltage in this manner, the transducer does not experience the effects of $C_{P}$, and the extracted power reaches its peak at the resonant frequency when the inductor ($L_{s}$) resonates with the capacitor ($C_{s}$).

These SSH techniques offer significant improvements in harvested power compared to traditional rectifiers. However, they require precise switching and control circuits and may face challenges during cold start-up. Incorporating a passive charging path provides a common solution to address this issue.

### 4.1. Open-Circuit Interface Circuits

#### 4.1.1. Synchronous Electric Charge Extraction (SECE)

**Figure 5:**
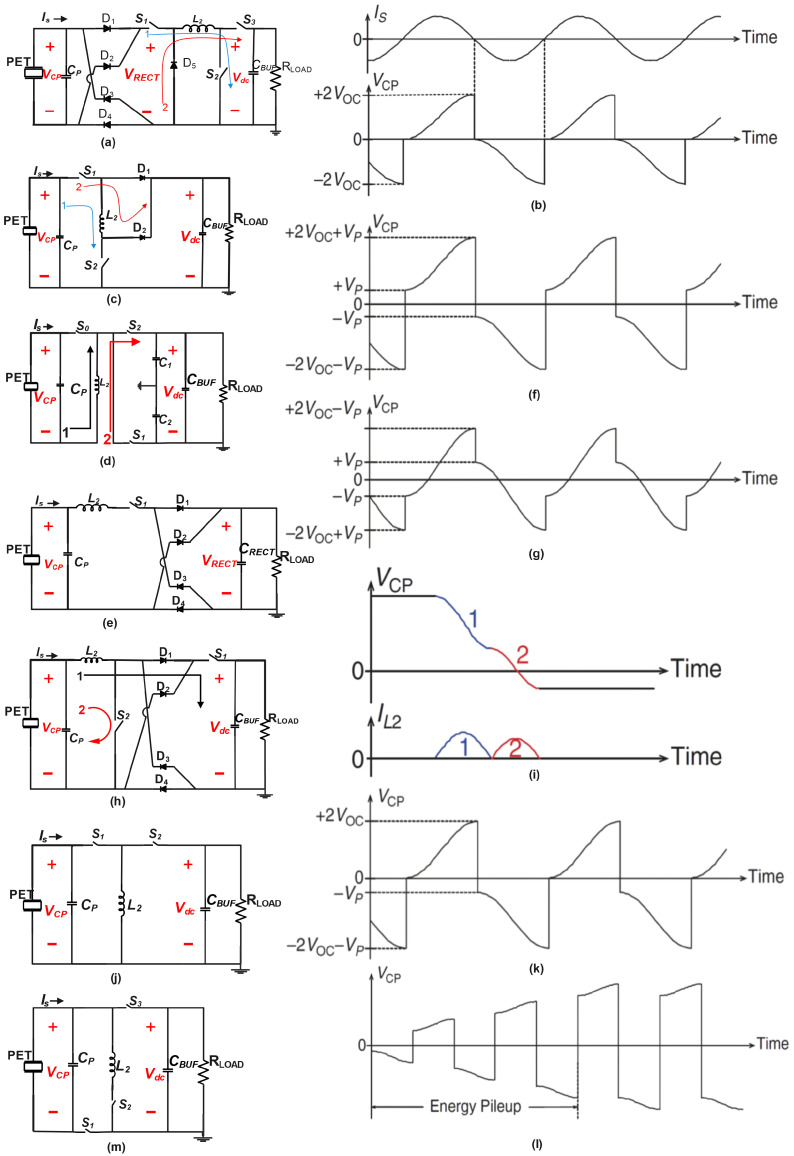
The open-circuit interface circuits: the schematics and waveforms of (**a**) a conventional SECE schematic, (**b**) conventional SECE waveforms, (**c**) a rectifierless SECE schematic, (**d**) another rectifierless SECE schematic, (**e**) an S-SSHI schematic, (**f**) a predamping waveform, (**g**) a less-damping waveform, (**h**) a two-step S-SSHI schematic, (**i**) two-step S-SSHI waveforms, (**j**) an energy-investing schematic, (**k**) an energy-investing waveform, (**l**) an energy pileup waveform, and (**m**) an SA-SSH schematic.

SECE, an open-circuit interface operation, was first introduced in [4]. In the basic SECE architecture (Figure 5a,b), the transducer is connected to a full-bridge rectifier (FBR), two switches ($S_{1}$ and $S_{2}$), and an inductor ($L_{2}$) in series. Unlike the FBR architecture, SECE lacks a capacitor at the rectifier’s output. Instead, a buffer capacitor ($C_{BUF}$) is connected in parallel with the effective loading from the application circuits ($R_{LOAD}$). It is linked to $L_{2}$ using another switch ($S_{3}$) and a diode ($D_{5}$).

During operation, the switches remain mostly off while $I_{s}$ charges and discharges ($C_{P}$) in an open-circuit manner. Similar to SSD-S operation, SECE nullifies ($V_{C_{P}}$) the voltage across $C_{P}$ by activating $S_{1}$ and $S_{2}$ when the current through the transducer $I_{s}$ reaches zero crossings. However, instead of dissipating electrical energy as heat, SECE converts potential energy into magnetic energy in $L_{2}$ by turning on the switches for a controlled duration. After disconnecting $L_{2}$ from the transducer by deactivating $S_{1}$ and $S_{2}$, another operation activates S3, converting the magnetic energy of the inductor into energy stored in $C_{BUF}$.

Figure 5b illustrates the waveform, indicating that $V_{CP}$ is charged from 0 V to $2V_{OC}$ during the positive current cycle. This implies that $0.5C_{P}{\left(2V_{OC}\right)}^{2}$ potential energy can be harvested during each switching activity, resulting in four times higher extracted power ($4C_{P}V_{OC}^{2}f$) compared to an FBR or HBR. Despite the power consumed by active switching and the degradation of extracted power, the additional harvested power outweighs the power cost. In a specific implementation, a control circuit consumes 300 μW, while the extra harvested power is 3.85 mW [22].

In another implementation illustrated in Figure 5c, the rectifier is eliminated, resulting in a rectifierless SECE. During the switching activity, energy in $C_{P}$ is transferred to $L_{2}$ by activating $S_{1}$ and $S_{2}$. In the subsequent operation, one of the switches is deactivated based on the signal polarity, and either $D_{1}$ or $D_{2}$ provides the current path to convert the inductor energy into energy stored in $C_{BUF}$. This implementation eliminates rectifier losses and achieves a mechanical-to-electrical conversion efficiency of 78% [23]. However, it has the limitation of negative node voltages during operation, and D2 must remain off during positive current cycles, which requires the open-circuit voltage ($V_{OC}$) to be lower than the DC voltage ($V_{dc}$). This issue can be addressed by applying dynamic body biasing on S1.

Shareef et al. proposed a different rectifierless SECE implementation (Figure 5d) using two capacitors ($C_{1}$ and $C_{2}$) for positive and negative cycles, achieving a conversion efficiency of 73% [24]. Nonetheless, this operation also results in negative voltages, requiring negative supplies.

To reduce conduction losses in switches, the current during energy transfer can be decreased by increasing the inductance of $L_{2}$. Gasnier et al. introduced multishot SECE operation, where potential energy in $C_{P}$ is converted into $L_{2}$ in multiple steps, reducing the peak inductor current and associated conduction losses. This design extracts 25% more power compared to traditional SECE [25].

SECE offers the advantage of decoupling the interface operation from the load voltage $V_{dc}$. This is possible because $C_{P}$ is always completely discharged in SECE, resulting in an extracted power of $4C_{P}V_{OC}^{2}f$. However, SECE rarely harvests the full available power of the transducer due to specific conditions [26].

#### 4.1.2. Predamping

The power extracted by the Synchronous Electric Charge Extraction (SECE) technique scales with the square of the open-circuit voltage ($V_{OC}^{2}$) and maximizes at a power point ($P_{AVL}$) when $V_{OC}$ equals $\frac{r}{16}V_{S}$. However, typical piezoelectric transducers exhibit a $V_{OC}$ significantly lower than $\frac{r}{16}V_{S}$ [26]. While the full-bridge rectifier (FBR) charges the piezoelectric capacitor ($C_{P}$) from −$V_{RECT}$ to +$V_{RECT}$, the SECE charges it from 0 V to 2$V_{OC}$. Employing synchronous switching, it is feasible to precharge $C_{P}$ to a voltage ($V_{P}$) exceeding 0 V immediately prior to the positive current cycle. This precharging method is termed “predamping” in the literature [27].

**Figure 6:**
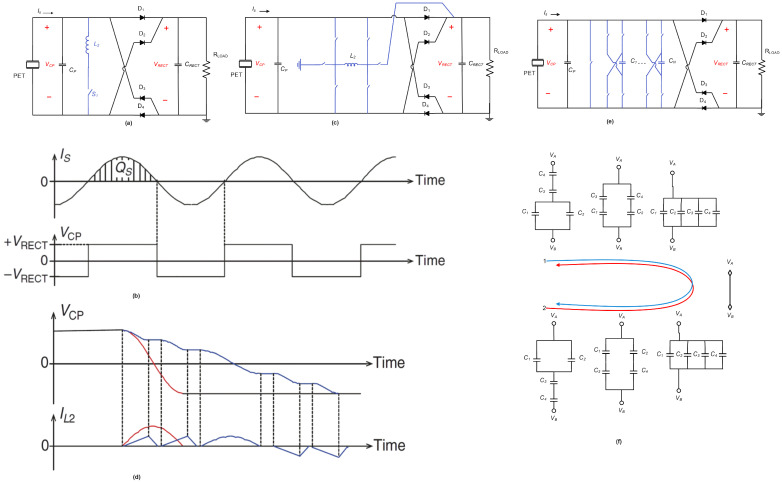
The short-circuit interface circuits. Schematics and waveforms of (**a**) a P-SSHI schematic, (**b**) P-SSHI waveforms, (**c**) a multistep P-SSHI schematic, (**d**) multistep P-SSHI waveforms, (**e**) a P-SSHC schematic, and (**f**) a FCR operation.

An example of a predamping interface is the series synchronized switch harvesting on inductor (S-SSHI) circuit [26], illustrated in Figure 5e–g. This circuit resembles those in Figure 6a,b, but with the FBR and switch+$L_{2}$ positions interchanged. This maintains the same effective series combination and eliminates the need for two pads for the external inductor, while $V_{RECT}$ remains dc in this particular configuration. During the positive peaks of $V_{CP}$, switch $S_{1}$ is activated, transferring energy from $C_{P}$ to $C_{RECT}$ via $L_{2}$. As $V_{CP}$ falls below $V_{RECT}$, the current through $L_{2}$ diminishes, while $C_{P}$ continues to discharge. Unlike the SECE, which deactivates $S_{1}$ upon complete discharge of $C_{P}$, this method deactivates $S_{1}$ when the inductor current reaches zero, thus negating the requirement for a de-energizing diode $D_{5}$.

When $V_{OC}$ exceeds 2$V_{RECT}$, this process can discharge $C_{P}$ to a negative voltage ($-V_{P}$), as depicted in the waveforms of Figure 5f. This results in a positive predamped voltage ($V_{P}$) before the onset of the positive current cycle. The extracted power can be determined using equation $\left(\left(4V_{OC}^{2}C_{P}+4V_{OC}C_{P}V_{P}\right)\cdot f\right)$. Notably, when $V_{P}$ = 0, the output power is equivalent to that of the SECE. However, if $V_{OC}$ is less than or equal to $V_{RECT}$, with $L_{2}$ connected between the transducer and $C_{RECT}$, $V_{CP}$ remains above 0 V after the discharging switching event, preventing the intended “predamping” effect. In [6,28,29], a two-step process is introduced, incorporating an additional voltage inversion after energy extraction. Figure 5h,i depict a specific implementation [6] utilizing a single inductor. The additional voltage inversion is facilitated by switch $S_{2}$, achieving the desired predamping. Furthermore, the energy extraction switch $S_{1}$ is positioned after the rectifier, simplifying the implementation as $S_{1}$ operates under a dc voltage.

Since S-SSHI shares the same circuit architecture as the SECE and differs only in the duration of $S_{1}$ and $S_{2}$ being turned on, predamping can be applied to various implementations of SECE by extending the ON time of $S_{1}$ and $S_{2}$. Yang [27] reported an output power that is 7.8 times higher than that of an FBR using the same architecture as shown in Figure 5d.

It is worth noting that predamping by discharging $C_{P}$ to a negative voltage ($-V_{P}$) may not always improve performance. In some systems, predamping can lead to overdamping [21], and in certain cases, ‘’less damping” is preferable with a $V_{CP}$ waveform as shown in Figure 5g is desired [6].

#### 4.1.3. Energy Investing or Pileup

Building upon the earlier discourse regarding predamping, we now delve into a related concept put forth in [3]. The proposed approach involves an energy-investing operation, which is visually represented in Figure 5j,k. When $V_{CP}$ reaches its positive peaks, $V_{dc}$ is briefly connected across $L_{2}$ before $C_{P}$ is discharged. This initial connection sets an inductor current, effectively investing some energy. Then, $L_{2}$ is connected to $C_{P}$, and the nonzero initial current helps drain $C_{P}$ to a more negative voltage when the inductor current drops to zero. This boosts the damping and the extracted energy. It is important to note that the proposed operation is asymmetric, as energy is extracted only at negative $V_{CP}$ peaks, and it requires only two switches. However, the invested energy comes from the energy buffer, undergoing two additional transfers and associated losses compared to the harvested energy. Although the control circuit consumes only 630 nW, the reported conversion efficiency is 69%, and the maximum output power increasing rate is 247% [3]. This segment delves into advanced techniques that enhance power extraction through dynamic switching strategies. The summary recaps how methods like Synchronous Electric Charge Extraction (SECE), various SSHI schemes, predamping, and energy investing effectively overcome the limitations of conventional rectifiers, despite introducing increased design complexity and demanding precise control.

In addition to the previously discussed techniques of predamping and energy investing, the authors of [30,31] introduce another innovative method known as energy pileup. This concept is illustrated in Figure 5l. In the energy pileup mode, the design initially activates $S_{1}$ when $V_{CP}$ reaches its peak. By carefully controlling the ON time, this operation achieves voltage inversion through $L_{2}$ without extracting energy, allowing $V_{CP}$ to gradually accumulate. Once $V_{CP}$ reaches the desired value, typically constrained by the breakdown voltage of interface transistors, the operation transitions to the conventional S-SSHI energy transfer mode. Remarkably, the design presented in [30] demonstrates a significant increase in the maximum output power rate, reaching up to 422% without the losses associated with energy investment.

Furthermore, Chamanian et al. [32] propose a self-adapting Switched-Source Half-Bridge (SA-SSH) interface that utilizes only three switches, as depicted in Figure 5m. This design performs energy pileup at positive peaks and energy extraction as well as investment at negative peaks. With this asymmetric operation, a more symmetric waveform can be maintained, reducing concerns about mechanical stability. An extracted power five times higher than that of an FBR is reported. In [33], a thermoelectric generator (TEG) is combined with the piezoelectric transducer to accelerate the energy pileup process.

### 4.2. Short-Circuit Interface Circuits

In the previous section, we discussed the open-circuit type of SSH (Switched-Source Half-Bridge) interface, where energy extraction occurs only during short periods at zero crossings and voltage peaks. Now, we cover the second type of the SSH interface, known as the short-circuit type, which enables energy transfer for most of the cycle. By employing synchronized switching techniques, the energy transfer duration is maximized, leading to higher extracted power.

#### 4.2.1. Utilizing Inductors

One well-known short-circuit interface operation is called P-SSHI or bias-flip interface. In this operation, the voltage inversion across the energy storage capacitor ($C_{P}$) is accelerated using a synchronized switched inductor, resulting in faster alternating path activation in the rectifier. This allows the transducer to primarily experience the effective loading through the rectifier, bypassing $C_{P}$. The extracted energy per cycle in this operation can be calculated as 2$Q_{S}V_{RECT}$, where $V_{RECT}$ replaces the open-circuit voltage ($V_{OC}$) in the SECE case, as shown in Figure 6a,b. By tuning $V_{RECT}$ to $V_{S}$/2, maximum power transfer (MPT) can be achieved. To improve the voltage inversion process, larger inductors are often used to reduce the inversion current and associated losses. A multistep P-SSHI design has also been proposed to enhance inversion efficiency; Figure 6c,d.

In P-SSHI, proper setting of $V_{RECT}$ is crucial for extracted power optimization. To achieve this, a switching dc–dc converter operating in discontinuous conduction mode (DCM) is commonly employed. This converter not only transfers the extracted energy to storage but also adjusts the effective loading by setting $V_{RECT}$, enabling MPT. A control scheme has been proposed to share the inductor for both interface bias flipping and the dc–dc converter, reducing the form factor [1].

#### 4.2.2. Inductorless Approaches

To reduce the size of the interface, an inductorless approach called P-SSHC has been proposed. This approach utilizes capacitors to achieve voltage flipping without the need for a large inductor [34]. Multiple capacitors with the same capacitance as $C_{P}$ are connected in parallel across the interface via switches. During the voltage-flipping process, the switches control the progressive sharing of charges between $C_{P}$ and the capacitors, effectively reducing $V_{CP}$. The capacitors are then connected back to $C_{P}$ with the opposite polarity to increase $V_{CP}$ in the opposite direction as shown in Figure 6e. This inductorless design achieves a flipping efficiency of 80%, but the required capacitors are currently too large for on-chip integration.

In a split-electrode piezoelectric transducer, implemented by Du and Seshia, on-chip capacitors are used in P-SSHC [35]. The electrodes of the transducer are reconfigured to enable series or parallel connections, reducing the capacitance to be inverted during voltage flipping. Another switching scheme called flipping-capacitor rectifier (FCR) utilizes on-chip capacitors configured in series and parallel to achieve efficient voltage flipping [36]. This scheme achieves a flipping efficiency of 85%. Further improvements have been made in capacitor switching algorithms to reduce intrinsic losses and enable the shared use of capacitors for interface bias flipping and the dc–dc converter.

Additionally, an experimental approach called ‘’SSH on an oscillator” has been proposed by Lallart et al. [37], where $C_{P}$ is connected to a second high-frequency piezoelectric oscillator during voltage flipping; Figure 6f. This triggers a high-frequency mechanical vibration, enhancing the voltage inversion process. This design offers an inductorless and potentially more economically viable solution for SSH interfaces.

Focusing on short-circuit approaches such as P-SSHI and integrated MPPT strategies, this section illustrates how these circuits maximize energy transfer by extending the energy extraction window. The summary emphasizes the balance achieved between improved efficiency and the challenges of implementing synchronized switching under varying load and frequency conditions.

#### 4.2.3. Maximum Power Point Tracking

Among the discussed interface circuits, the SECE circuit stands out as the only one where output power depends solely on $V_{OC}$ (open-circuit voltage). In SECE, the energy in $C_{P}$ is harvested completely whenever its voltage peaks. This decouples energy harvesting from the load condition, and the output power is determined by $V_{OC}$, which is influenced by vibration, mechanical parameters, and $C_{P}$.

On the contrary, in all other interface circuits, the extracted power relies on specific voltages such as $V_{CP}$. These voltages, like the amplitude set by $V_{RECT}$ in FBR/HBR and short-circuit interfaces or the predamping voltage VP in open-circuit cases, depend on the effective load $R_{LOAD}$. A switching dc–dc converter in the discontinuous conduction mode (DCM) is often used to emulate a resistive load. The effective $R_{LOAD}$ and resulting $V_{RECT}$ can be adjusted by altering the switching timing. For instance, the output power of FBR is given by the expression 4$C_{P}V_{RECT}\left(V_{OC}-V_{RECT}\right).f$, which reaches its peak when $R_{LOAD}$ is set such that $V_{RECT}$ = $V_{OC}/2$. Due to this dependency, an MPPT (Maximum Power Point Tracking) circuit is required for all interface circuits except SECE.

**Figure 7:**
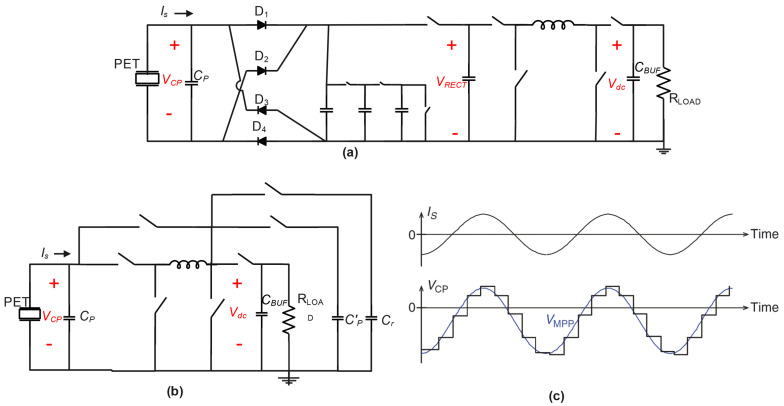
The schematics and waveforms of (**a**) a $V_{OC}$ sensing schematic [38], (**b**) a sense-and-set schematic [39], and (**c**) sense-and-set waveforms.

MPPT circuits can be broadly categorized into two types: (1) open-loop “fractional $V_{OC}$” methods and (2) closed-loop approaches like “P&O”. In fractional $V_{OC}$ techniques, $V_{OC}$ is periodically measured by disconnecting the interface circuit from the transducer, as shown in Figure 7a. A passive or active peak detector is used to sense $V_{OC}$, and techniques like voltage division or charge sharing generate the desired fractional voltage for reference. The switching timing of a subsequent buck–boost dc–dc converter is then adjusted to achieve MPPT. On the other hand, closed-loop methods continuously measure the output power while the harvester is connected to the transducer. Algorithms such as hill climbing are used to adjust operating parameters for maximizing the output power within the measurement tolerance. MPPT circuits perform two tasks: sensing and tuning.

In the fractional $V_{OC}$ method, $V_{OC}$ is sensed periodically with a low duty cycle by disconnecting the harvester from the transducer [12,38]. Passive or active peak detectors are used, and techniques like resistive ladder voltage division or charge sharing are employed to generate the desired fractional voltage. The switching timing of a subsequent buck–boost dc–dc converter is adjusted accordingly to set $V_{RECT}$ to the desired fractional voltage, achieving MPPT [38]. Various examples of this method have been proposed, demonstrating high power-harvesting efficiency and fast tracking times.

Another approach, the ‘’sense-and-set” method [12], oversamples the current ($I_{S}$) and calculates the instant $V_{MPP}$ using a mixed-signal circuit. IS is measured by charging $C_{P}$ for a short period, and the resulting small voltage is amplified and transferred to a capacitor to indicate IS. Combined with high-frequency open-circuit interface operation, this method maintains $V_{MPP}$ dynamically across $C_{P}$, resulting in improved power extraction compared to an FBR. This approach showcases the potential of advanced IC technology in implementing complex control schemes to enhance power extraction for vibrational energy harvesting.

#### 4.2.4. Perturb and Observe (P&O)

The Perturb and Observe (P&O) method is a closed-loop MPPT technique that aims to maximize the output power by directly measuring a physical quantity that indicates the extracted power. One implementation of P&O involves using a flyback converter with constant ON time modulation, where the peak inductor current is measured through a small sensing resistor to determine the extracted power. The sensing voltage is digitized using an analog-to-digital converter, and a digital circuit in a low-power microcontroller unit (MCU) implements the P&O algorithm. This approach has shown an MPPT efficiency above 94% [13].

Another fully mixed-signal implementation evaluates the extracted power by multiplying the converter’s input voltage and current [40]. By biasing the MOSFET in the subthreshold region to exploit logarithmic current–voltage characteristics, the power level indication is obtained through current summation. This design achieved an end-to-end efficiency peak of 88% and an MPPT peak efficiency of 99.8%. While the mentioned implementations are suitable for electrostatic energy harvesting, they can be applied to piezoelectric energy harvesting as well.

#### 4.2.5. Nonresonant Operation

The discussed interface circuits primarily focus on achieving resistive loading, assuming that the impedance of a piezoelectric cantilever vibrating near its resonant frequency is mostly resistive [7,13]. However, in real-world applications like infrastructure or factory monitoring, the operating frequency may deviate from the piezo structure’s resonant frequency. This frequency offset leads to a reactive impedance that cannot be effectively matched with a purely resistive load, resulting in a significant degradation of the extracted power. To address this issue, Hsieh et al. proposed a method to achieve complex impedance matching by introducing a delay in the conventional switching methods [7]. By creating a phase offset between the interface voltage and current, the interface circuit presents a complex loading. The resistive part extracts power from the source, while the reactive part cancels the residual reactance of the piezoelectric structure. Adjusting the equivalent loading through variations in $R_{LOAD}$ and switching timing optimizes the extracted power. This intentional delay can be incorporated into both open-circuit and short-circuit interface types, improving harvesting performance when operating off-resonance. Experimental results have shown significant bandwidth extensions and improved power extraction efficiency [8,41,42,43].

#### 4.2.6. Charging Efficiency Analysis

The charging efficiency of piezoelectric energy harvesting varies significantly among different interface circuits, ranging from basic to advanced topologies. Standard full-bridge rectifiers offer simplicity but limited efficiency (20–30%), while voltage doublers achieve slightly higher efficiency (30–40%) with increased output voltage. Advanced techniques such as Synchronized Switch Harvesting on Inductor (SSHI) demonstrate marked improvements, with parallel SSHI reaching 40–60% efficiency and series SSHI achieving 50–70%. The Synchronized Electrical Charge Extraction (SECE) technique shows the highest efficiency (60–80%) and offers load-independent operation, though with increased implementation complexity. Charging efficiency is influenced by multiple factors, including circuit parameters, operating conditions, and system integration aspects. Optimization strategies focus on component selection, control methods, and system design considerations such as impedance matching and parasitic minimization. The selection of interface circuits should balance efficiency requirements against implementation complexity and cost constraints, considering specific application needs in self-powered systems.

## 5. Self-Powered Systems—SPSs

Among the aforementioned studies, it remains unclear whether current independent systems that can operate autonomously without external energy inputs. The subsequent discussion focuses on such systems and their respective output levels based on their energy requirements. These self-powered systems can be broadly categorized into two distinct types: simple self-powered systems (Single SPSs) and array self-powered systems (array SPS).

### 5.1. Single SPS

**Figure 8:**
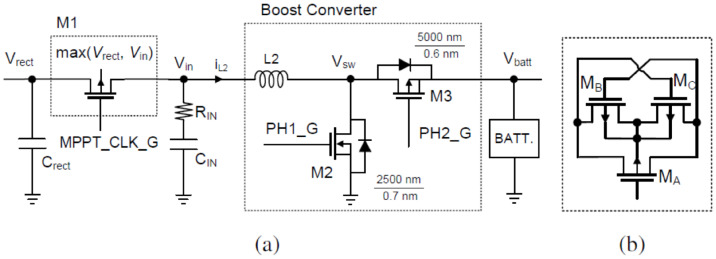
Boost converter (**a**) with its interface; (**b**) implementation of M1 [44].

In [44], an innovative self-powered piezoelectric energy harvesting circuit is introduced that combines a maximum power point tracking (MPPT) circuit with a synchronized switch harvesting on inductor (SSHI) circuit (see Figure 8). The integration of these circuits enhances energy harvesting efficiency over a wide range of power levels from the piezoelectric transducer. The proposed circuit achieves an impressive peak efficiency of 77% and can harvest energy within a power input range of 10 μW to 34 μW during MPPT operation. It features three operation modes that expand the range of harvestable power levels, increasing the versatility of the system. The circuit is designed with robustness in mind, employing suitable MOSFETs and carefully selected parameters for reliable operation. Notably, it can cold-start even when the capacitor and battery are fully discharged, ensuring autonomous and self-sustaining operation. Experimental results demonstrate the circuit’s superior performance compared to competing designs, particularly in terms of its wider range of harvestable power levels and higher efficiency. The proposed circuit has significant implications for various applications, including wireless sensor networks, wearables, and IoT devices, as it reduces reliance on batteries and external power sources. Future research directions include exploring different operating conditions, integrating hybrid energy sources, optimizing power consumption, and establishing standardized testing protocols for accurate comparison and benchmarking. Addressing these areas will further enhance the field of piezoelectric energy harvesting and advance the proposed circuit’s performance and applicability. The paper’s limited scope of comparison, focusing primarily on recent piezoelectric energy harvesting circuits with MPPT, could be broadened to include a more comprehensive comparison with other energy harvesting techniques like solar, thermoelectric, or electromagnetic harvesting. A more detailed analysis of the proposed circuit’s performance in different scenarios, including variations in environmental conditions and vibration frequencies, would provide a better understanding of its robustness. Exploring the generalizability of the circuit by considering various transducer configurations, materials, and practical constraints would further enhance its evaluation. Additionally, conducting a detailed analysis of the power consumption of the control circuitry and exploring methods for reducing it would improve overall system efficiency. The lack of real-world application validation could be addressed by including practical implementation examples or case studies to demonstrate the circuit’s effectiveness in specific applications.

**Figure 9:**
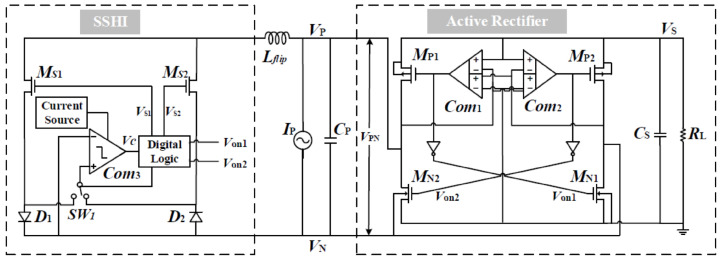
Simplified diagram of the proposed self-powered SSHI interface circuit [45].

A novel self-powered piezoelectric energy harvesting interface circuit is incorporated beside the active rectifier, an adaptive synchronized switch harvesting on inductor (SSHI) circuit. The circuit efficiently harvests and converts electrical energy from ambient vibrations into a stable output voltage. Unlike traditional SSHI circuits, this circuit eliminates the need for external control by utilizing the output voltage of the comparator in the active rectifier as the trigger signal for SSHI, allowing it to adapt to varying inductance. The incorporation of a rail-to-rail hysteresis comparator fine-tunes the voltage flipping process and reduces static power consumption, resulting in improved power efficiency. Moreover, the circuit exhibits self-powered cold-start capability, making it suitable for remote or inaccessible locations. Simulation results demonstrate a significant 4.6-fold enhancement in energy extraction efficiency compared to a classical full-bridge rectifier circuit. The low power consumption of the control part further reinforces the circuit’s efficiency. The proposed circuit contributes to the field of piezoelectric energy harvesting by offering improved energy extraction, reduced static power consumption, and self-sustaining power generation. It has broad applications in low-power electronic devices like wireless sensor networks, wearables, and IoT devices. However, it is essential to consider potential limitations and biases in the study, such as the need for further optimization and testing to ensure real-world performance, as well as the circuit’s compatibility with different configurations and technologies [45] (see Figure 9).

The proposed circuit lacks real-world validation and has limited experimental testing, as simulation results may not fully capture practical challenges in energy harvesting applications. The circuit’s generalizability may also be questioned, considering its optimization for specific conditions and materials, potentially limiting its applicability to different scenarios or piezoelectric materials. Additionally, scalability, complexity, and practical feasibility in larger power generation applications should be addressed, along with considerations of reliability, robustness, and long-term durability. A paper could further explore power management strategies and load matching techniques to optimize energy utilization and adapt to varying load conditions or changes in energy availability [45].

The discussion on self-powered systems outlines the transition from individual to array-based architectures, showcasing the potential of autonomous energy harvesting systems. This summary captures the benefits of reduced external dependency and highlights remaining challenges, such as scalability, long-term reliability, and the need for robust experimental validation in diverse operational environments.

**Figure 10:**
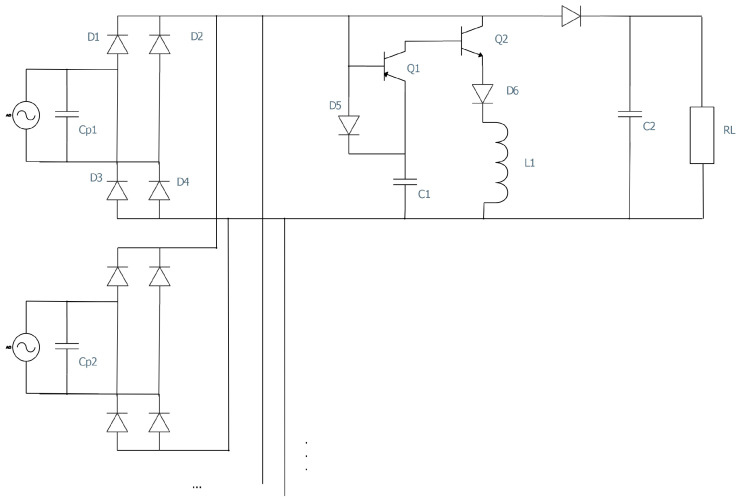
Proposed self-powered multi-input P-SSHI [46].

The self-powered rectifierless synchronized switch harvesting on inductor (ReL-SSHI) circuit introduces a revolutionary approach to piezoelectric energy harvesting [46]. It operates in two modes, eliminating the need for a rectifier bridge and optimizing energy transfer. In the first mode, efficient energy exchange occurs between capacitors and inductors during the LC resonance period. The second mode enhances energy harvesting by reciprocally exchanging energy between capacitors. By bypassing the limitations of traditional rectifier bridge circuits, the ReL-SSHI circuit enables direct energy transfer and maximizes the utilization of piezoelectric energy. However, the circuit’s limited scalability for larger projects and high-demand applications could be a concern, as well as its reliance on ambient vibrations, which may compromise its effectiveness in low-vibration environments. Additionally, these issues highlight the complex implementation and integration challenges, potentially increasing costs and design complexity. Furthermore, critics question the circuit’s performance variability and compare it to alternative energy harvesting technologies, raising doubts about its efficiency, cost-effectiveness, and ease of implementation [46] (Figure 10).

**Figure 11:**
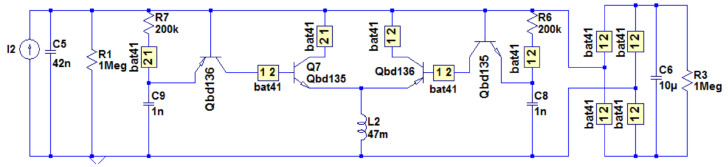
Optimized P-SSHI circuit using low-power components [20].

An improvement to the circuit by evaluating different switching techniques and proposing an optimization that greatly increases power output is reported in [20] (see Figure 11). By utilizing bipolar transistors in the switching technique, the circuit achieves a 19.2-fold higher power output compared to MOSFET-based switches. The optimization process involves replacing energy-consuming components with more efficient alternatives, resulting in an over 300% increase in power output. Experimental results validate the effectiveness of the optimization, demonstrating a high level of agreement with simulations. The self-powered P-SSHI circuit has applications in self-powered wireless sensor systems for various environments. The lack of detailed information regarding specific low-energy consumption components proposed in the paper complicate replication, and accurately evaluating the study is challenging. Additionally, the work lacks details about the experimental setup, conditions tested, and specific applications of the self-powered P-SSHI circuit, restricting the generalizability and applicability of the research.

### 5.2. Array SPS

#### Multiple  Inputs

Some works have explored interface circuits capable of working with multiple piezoelectric transducers, offering unique features not feasible with a single transducer system. For instance, using multiple transducers with different orientations enables energy harvesting from vibrations along different directions [9,47]. The inclusion of multiple electrodes in a disk-shaped piezoelectric transducer allows energy harvesting from random vibrations and various vibrational modes. Another approach involves adopting multiple transducers with different resonant frequencies to extend the operating frequency range. However, utilizing multiple transducers requires multiple interface circuits, which increases system complexity. To mitigate this, time-division multiplexing is preferred, and trade-offs need to be made regarding shared components, control schemes, and costs. For example, a wearable energy harvester with multiple flexible thin-film beams presented a time-interleaving interface that supported up to six transducers, enabling energy harvesting from multiaxial body motion [10].

A potential solution for piezoelectric energy harvesting is the self-powered multi-input bridgeless series SSHI circuit. This circuit integrates a voltage doubler topology, simplifies complexity, and maximizes power availability. It achieves a threefold increase in power harvesting by harnessing the energy from two PE transducers simultaneously. The circuit’s autonomous operation enhances reliability, and comparison tests confirm its superiority over alternative configurations. The implications of this research are vast, with applications in various fields. Further exploration should include validating performance under different conditions, designing a low-power maximum power point tracking circuit, and evaluating scalability across transducer types. Field tests and ongoing research on materials and methods will advance piezoelectric energy harvesting. In conclusion, this circuit represents a remarkable advancement, reshaping energy harvesting systems and driving sustainability and innovation.

**Figure 12:**
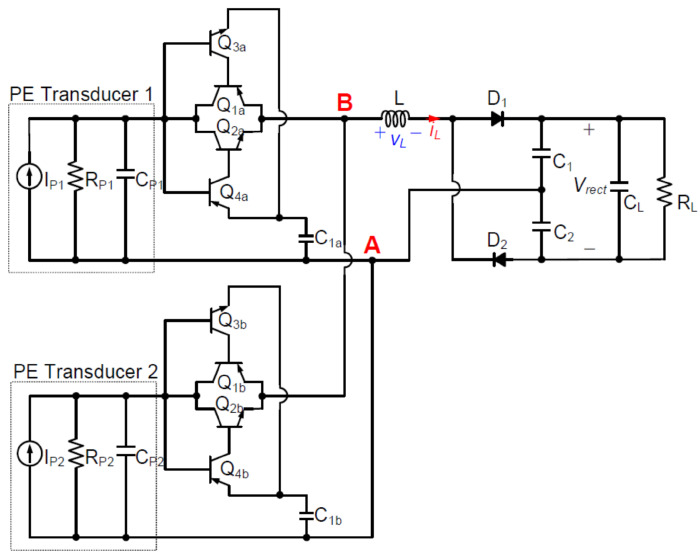
The proposed multi-input bridgeless S-SSHI circuit for PE energy harvesting [19].

The research presented in [19] (see Figure 12) focuses on a specific circuit design for piezoelectric energy harvesting, namely a self-powered multi-input bridgeless series SSHI circuit. While this circuit design shows potential, it is important to acknowledge its limited scope. The text lacks a comprehensive comparison with existing circuits or methodologies. Thorough comparative analysis with other state-of-the-art approaches would shed light on the proposed circuit’s performance and potential advantages or disadvantages. Moreover, the text does not provide sufficient experimental details, such as the experimental setup, measurement procedures, or statistical analysis; the replicability and reliability of the results is unconfirmed and there is lack of uncertainty assessment. To validate the findings, it is crucial to provide comprehensive information regarding the experimental conditions, allowing others to replicate and verify the results. Furthermore, the laboratory-based experiments may not fully capture the challenges and complexities of practical implementation. Evaluating the circuit’s performance in real-world scenarios considering varying environmental conditions and long-term durability is necessary to assess its practical viability. In terms of scalability and adaptability, the text lacks extensive information. While the proposed circuit design targets multiple piezoelectric transducers, it does not thoroughly explore its scalability or adaptability to different transducer configurations. The circuit’s performance may vary for different setups, emphasizing the need for further research to assess its versatility and robustness. Lastly, the reproducibility and generalizability of the study may be questioned due to the lack of detailed information about the experimental setup, measurement procedures, and specific component choices. Critics may argue that providing additional documentation and details is essential to ensure that other researchers can replicate the results and build upon the findings.

**Figure 13:**
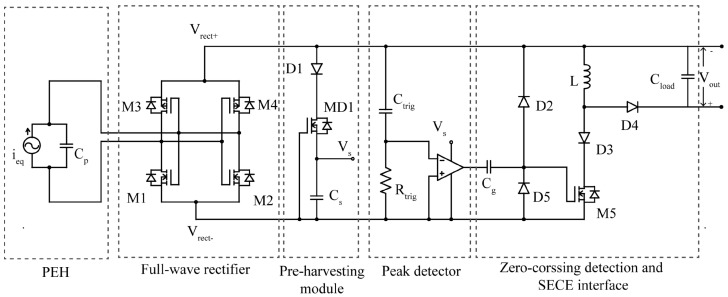
Extensible SECE rectifier proposed circuit [18].

By harnessing the power of two distinct piezoelectric (PE) transducers simultaneously, a circuit was developed to achieve a remarkable threefold increase in power harvesting under identical excitation conditions. The circuit’s design optimizes power availability by streamlining the resonant loop and minimizing the number of diodes. Furthermore, its ability to operate autonomously, even without an external power supply, enhances reliability and longevity. The proposed self-powered SECE interface circuit provides an efficient solution for harvesting energy from multiple piezoelectric transducers [18] (see Figure 13). It utilizes a passive zero crossing detection method and a single inductor to operate based on the SECE principle, ensuring load-independent power output. This guarantees stable and reliable power generation for various applications. SECE techniques, including the self-powered SECE interface circuit, offer several advantages in piezoelectric energy harvesting. Firstly, load independence ensures consistent power generation regardless of the connected load, enhancing reliability. Secondly, SECE techniques maximize energy harvesting capabilities by efficiently extracting and converting electrical charges from the transducers, optimizing energy harvesting from ambient vibrations. Moreover, SECE is well suited for multiple transducers, enabling higher power output and increased efficiency. Additionally, self-powered SECE interface circuits eliminate the need for extra sensors and external power supplies, simplifying system design and reducing costs. The SECE interface circuit can be employed with multiple piezoelectric energy harvesters, as the load, diode, and capacitor in the circuit can be shared among them. This enables efficient energy extraction from multiple sources, expanding the potential applications of the circuit. The provided references ([11–13]) in [18] offer further reading on energy harvesting, including studies on ambient vibrations, multi-input synchronous charge extraction circuits, and optimized self-powered switching circuits for non-linear energy harvesting.

The visualization figure of the circuit performance in the PDF file presents a comparison of the output power between the ZSECE circuit, full-bridge rectifier (SEH), and conventional self-powered SECE. This graph depicts the output power in micro-Watts (μW) for each of the mentioned circuits, providing a valuable insight into the capabilities and potential advantages of the self-powered SECE interface circuit.

However, with all these impacts, there is a lack of experimental validation. Without empirical data to support the claims, there may be skepticism regarding the actual performance and efficiency of the self-powered SECE rectifier. While theoretical descriptions and simulations are valuable, experimental evidence is crucial to demonstrate its effectiveness. Besides the limited scope of application, the circuit’s suitability might be restricted to specific scenarios or controlled environments. It becomes necessary to assess its adaptability and performance in real-world conditions beyond laboratory settings to determine its practicality. Additionally, the associated complexity and cost may make it less feasible for widespread adoption, particularly in applications that prioritize cost-effectiveness, Efficiency and trade-offs are additional concerns. Lastly, the absence of comprehensive comparative studies in the paper make the evaluation incomplete against existing energy harvesting technologies to determine its true value and advantages within the broader context of the field. Comparative studies can provide insights into its strengths and weaknesses and facilitate informed decision-making.

The interface circuit for multi-input piezoelectric energy harvesting addresses the challenge of phase mismatch among PZTs [17]. It features an improved rectifier circuit topology, self-powered strategy, and simplified structure, enabling efficient energy extraction from multiple sources while maintaining high performance and self-sustainability. The circuit utilizes a full-bridge rectifier to handle arbitrary phase differences, eliminating power loss. The self-powered strategy, called multi-input parallel synchronized switching harvesting on inductor (P-SSHI), maximizes energy utilization. The circuit has a streamlined design with fewer switches and inductors, achieving efficient multi-input energy harvesting. The output voltage waveform exhibits unique characteristics, including rise time and voltage fluctuation. A clamping mechanism maintains a specific voltage level during the supply stage.

When considering the proposed circuit [17], it is important to address various practical implementation challenges. One concern is the lack of thorough validation through real-world implementations, as the absence of empirical evidence and experimental results could question the circuit’s viability and practicality. Another area of scrutiny is the circuit’s scalability and robustness, particularly when dealing with a large number of input cells or variations in the phase difference. The complex scenarios could lead to diminished performance or increased complexity in practical applications.

Furthermore, a comparative analysis that evaluates the proposed circuit against existing state-of-the-art approaches and alternative circuit topologies are needed. Without such a comparison, the distinct advantages or disadvantages of the proposed circuit may not be fully understood. Also, potentially limiting its adoption in practical applications. The limited scope of the described circuit primarily focuses on addressing the phase mismatch problem in multi-input piezoelectric energy harvesting. It may be contended that other important aspects, such as system optimization, power management, or integration with other renewable energy sources have not been adequately addressed, potentially limiting the circuit’s overall effectiveness.

**Figure 14:**
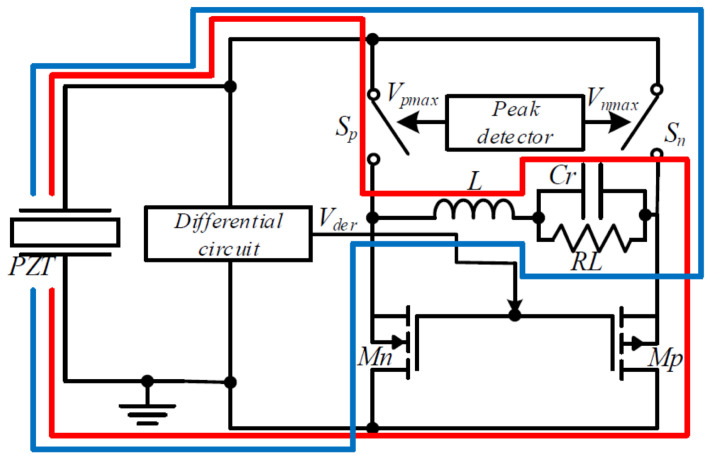
Topology of the SPEDS-SSHI circuit [16].

The Self-Powered Extensible S-SSHI circuit revolutionizes energy harvesting from multiple PZTs [16] (see Figure 14). Requiring no external power source, this novel circuit improves harvested power and offers great potential for enhancing energy efficiency. It harvests energy from multiple PZTs under a single vibration source, reducing the number of switches and enhancing component reuse. Simultaneous energy harvesting from multiple PZTs leads to higher energy output, better utilization of the energy source, and improved system reliability. Simulations confirm the circuit’s effectiveness in harvesting energy, eliminating energy loss caused by charge neutralization. The circuit’s ability to harvest energy from multiple PZTs with any phase difference sets it apart. However, limitations may include the lack of real-world experimental validation, limited comparisons with other circuits, and the need for impedance matching for optimal power output. The self-powering mechanism, the role of the RC differential circuit, and potential applications in various industries remain unaddressed. A novel circuit, referred to as SPEDS-SSHI, in [16] offers significant improvements in harvested power compared to traditional circuits by reducing the number of switches required and enhancing component reuse. What sets the SPEDS-SSHI circuit apart from existing piezoelectric energy harvesting interfaces is its self-powered nature. Unlike conventional circuits, it operates without the need for an external power source. Additionally, it can simultaneously harness energy from multiple PZTs with any phase difference, maximizing the electrical energy supplied to the load. By reducing the number of switches needed for a single piezoelectric unit and improving component reuse, this circuit design enhances cost-effectiveness and efficiency. To control the MOSFETs, the SPEDS-SSHI circuit employs an RC differential circuit, further optimizing the utilization of resources.

The advantages of simultaneous energy harvesting from multiple PZTs are numerous. Firstly, it leads to a higher total energy output compared to harvesting from a single PZT. By capturing energy from multiple PZTs operating under the same vibration source, the electrical energy supplied to the load experiences a significant boost. Moreover, this approach allows for better utilization of the available energy source. In real-world environments, PZTs may exhibit varying amplitudes and phases when driven by a single vibration source. By harvesting energy from multiple PZTs, the circuit can efficiently capture energy from each one, maximizing the overall energy harvesting potential. Finally, simultaneous energy harvesting from multiple PZTs enhances system reliability and stability. Distributing the energy harvesting load across multiple PZTs reduces dependence on a single unit, mitigating the risks of failure or performance degradation. The effectiveness of the SPEDS-SSHI circuit was verified through simulations using LTspice (version 17.0.37.0), confirming the theoretical analysis. The simulations considered PZTs produced by the same manufacturer, assuming identical internal parasitic parameters ($C_{P}$ and $R_{P}$) for each unit. Different vibration rates were reflected by varying ip values. Simulation results demonstrated the SPEDS-SSHI circuit’s prowess in harvesting energy from multiple PZTs. Regardless of the number of PZT cells utilized, the inductor current direction remained consistent, eliminating energy loss caused by neutralizing positive and negative charges, a common issue encountered in classical S-SSHI circuits. One noteworthy distinction of the SPEDS-SSHI circuit is its ability to simultaneously harvest energy from multiple PZTs with any phase difference. In contrast, classical S-SSHI circuits exhibit a fixed 180° phase difference between the positive and negative peak values of the currents. There are several important aspects to be aware of in [16]. Firstly, it is worth noting that the texts lack experimental validation, relying solely on theoretical analysis and simulations. While simulations provide valuable insights, experimental validation in real-world conditions would greatly enhance our understanding of the circuit’s performance and efficiency. Another point to consider is the limited scope of comparison provided. Although a comparison with existing S-SSHI circuits is mentioned, the specific circuits and their performance metrics are not provided. A more comprehensive comparison with other state-of-the-art circuits would enable us to better evaluate the advantages and disadvantages of the proposed SPEDS-SSHI circuit. Furthermore, it is important to recognize that practical implementation challenges may arise when applying the proposed circuit in real-world applications. Factors such as component availability, cost, size, and integration with other systems need to be carefully considered. These practical considerations play a significant role in determining the feasibility and suitability of the circuit for practical use.

### 5.3. Self-Hybrid Systems—SHSs

As explained in the following, hybrid approaches can be used to develop the power conditioning circuits/systems of PEH based on the independent design methods covered in the preceding parts.

#### 5.3.1. A. Capacitor–Inductor Interface Circuits

**Figure 15:**
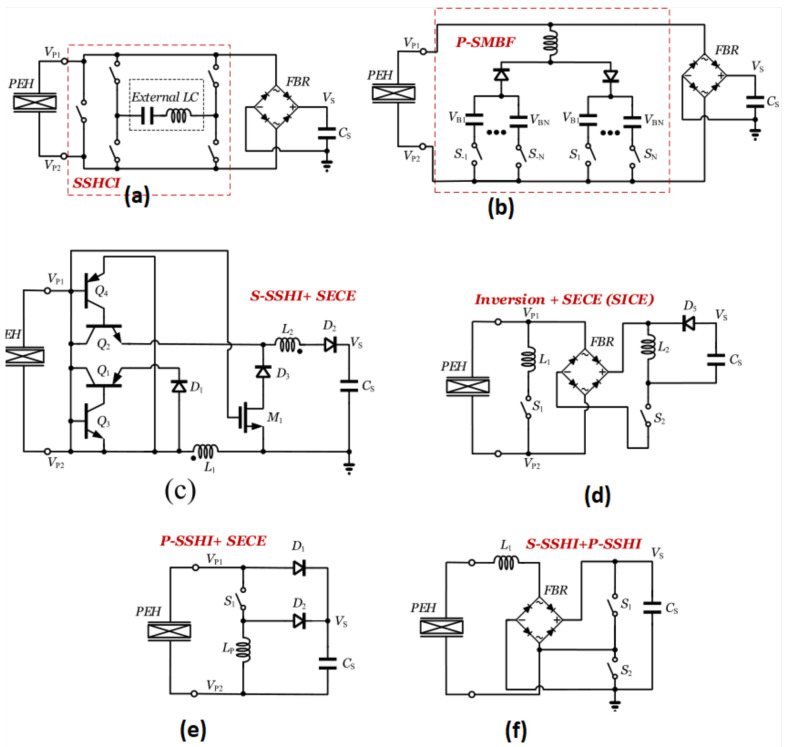
Hybrid inductor–capacitor energy harvesting [50]. (**a**) SSHCI [48]. (**b**) P-SMBF [51]. (**c**) S-SSHI+SECE [52]. (**d**) SICE [53]. (**e**) P-SSHI+SECE [52]. (**f**) S-SSHI+P-SSHI [54].

For the SSH process, a combination of inductor and capacitor can benefit from SSHI, SECE, and SSHC collectively to accomplish instantaneous voltage flipping, insensitivity to load, and compact design implementation, respectively. Çiftci et al. proposed in [48] to implement traditional SSHI using a small-sized off-chip inductor in series with an external capacitor to lower the inductor’s peak current creating a capacitor–inductor (SSHCI) interface circuit, as shown in Figure 15a.As a result, power loss during the $C_{P}$ voltage switching moments across component sizes may be significantly reduced. Using series bias voltages produced by auxiliary capacitors in series with the inductor, Liang [49] proposed the idea of multiple-bias flipping for parallel SSHI (see Figure 15b).

**Figure 16:**
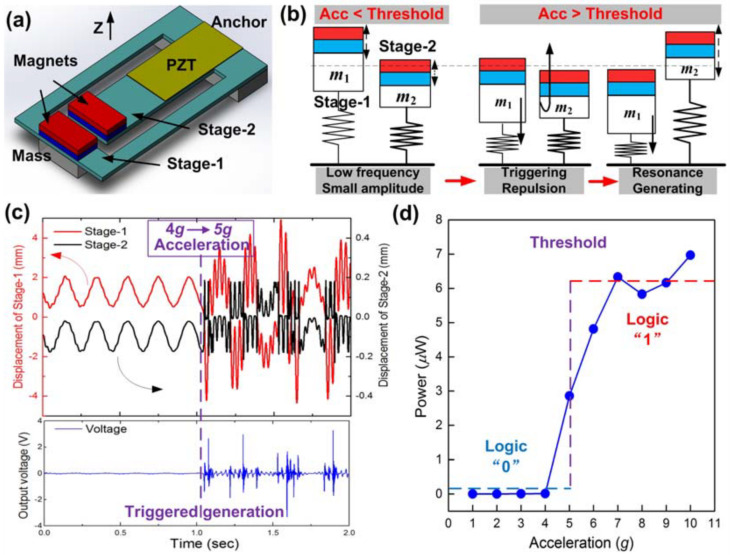
(**a**) Schematic and operation of the threshold-triggered energy harvester. (**b**) Working principle. (**c**) Simulation showing displacement and voltage output when acceleration increases from 4 g to the 5 g threshold at 1 s. (**d**) Threshold-triggered response, enabling a rapid switch from “0” to “1” like a logic circuit, functioning as a sensitive, power-free switch [55].

This innovative self-powered wireless sensing node utilizes a hybrid energy-harvesting mode to monitor and differentiate vibration patterns in the environment [55]. By combining dual-stage piezoelectric vibration energy harvesters and nonlinear electromagnetic harvesters, the node achieves self-sustainability and seamless wireless transmission of alarm signals. The dual-stage piezoelectric harvesters generate significant power when the ambient vibration meets predetermined acceleration and frequency conditions. Nonlinear electromagnetic harvesters enhance energy conversion efficiency and capture additional energy from low-acceleration and ultralow-frequency movements. The node’s comprehensive system includes sensors, signal-processing/submitting circuits, and wireless transmission components. Challenges such as inadequate on-site harvested electricity are overcome by the hybrid energy-harvesting mode, enabling the node to operate independently without relying on external power sources; Figure 16.

The lack of specific preset conditions for ambient vibration, such as acceleration and frequency, raises concerns among critics regarding the evaluation of the node’s effectiveness in different real-world scenarios [55]. Furthermore, the system’s limited scalability and adaptability in recognizing a broader range of vibration patterns may hinder its versatility and applicability [55]. The overall efficiency of the energy harvesting process highlights the need to optimize conversion efficiency and reduce energy losses within the system [55]. The node’s reliance on ambient vibrations as the primary energy source may face limitations in environments with limited vibration intensity or frequency, impacting reliability and operational lifespan [55]. Integration and miniaturization challenges arise when attempting to compactly integrate all the necessary components while maintaining optimal performance and energy efficiency [55]. Furthermore, the cost and feasibility of implementing the self-powered wireless sensing node on a large scale is an advantage, along with compatibility with existing infrastructure [55].

#### 5.3.2. B. Hybrid Inductor-Based Interface Circuits

This technique can be used to build a triple step for reversing the polarity of the $C_{P}$ voltage in SSHI using a single bias capacitor. Furthermore, by storing the collected energy and supplying the bias voltage needed for flipping, a storage capacitor is further utilized in [56] for multiple-bias flipping operation in a series SSHI interface circuit, simplifying the circuit design. Furthermore, the number of flipping cycles can be automatically changed based on the load’s condition. Chen and colleagues extended the multiple-bias flipping concept in [57] by incorporating a flipping time optimization process. If there was not enough room for the external inductor, this inductor–capacitor-based interface circuit may also be set up as a sole-capacitor-based one.

As covered before, SECE is one of the load-independent interface circuits that can greatly extend the output voltage’s range by first harvesting energy from an inductor and then delivering it to the load in two different loops. Consequently, Xia et al. [52] presented a self-powered hybrid series SSHI and SECE interface circuit [Figure 15c] which could use the electric charge extraction idea to achieve a stable equilibrium between load-decoupling and the maximum output power of the rectifier (obtained via SSHI).

In order to achieve this, BJT switches were used to identify the switching moments and set the circuit to either the SECE portion when the $C_{P}$ voltage flips in reverse or to the series SSHI component when it flips from positive to negative. As seen in Figure 15d, Lallart et al.’s [53] proposal included synchronized switch on inductor (SSI) and SECE, where SSI is equivalent to SSHI without the harvesting stage [58].

Instead, the harvesting stage in this method uses SECE. In order to appropriately raise the PEH voltage before energy extraction (SECE), n steps of inversion are required. An inducer harvests energy only once after the full inversion process is finished. As a result, the PEH voltage experiences a significant increase in amplitude prior to any charge extraction. As seen in Figure 15e, a different P-SSHI and SECE combination that is self-configurable based on the PEH’s OC voltage at very low vibrations was reported in [52]. With the rectified voltage of 4.14 V and the piezoelectric OC voltage of 3 V, the prototype circuit was able to extract a peak power of 85.7 μW. In terms of operational voltage range and peak output power, it outperformed FBR by more than two times. Moreover, Wu et al. presented a hybrid series/parallel SSHI interface circuit in [54] [Figure 15f], in which an inductor in the parallel SSHI loop was used to harvest electric charge. In order to transfer the scavenged energy to load and return to PEH, series SSHI was then constructed. When the OC voltage of the PEH was larger than the rectified voltage, this circuit configured itself similarly to synchronous inversion and charge extraction (SICE), acting as parallel SSHI and SECE in the positive and negative half-cycles of the PEH voltage, respectively. The range of the rectified voltage in series SSHI, which was previously constrained, was obviously enlarged by employing this hybrid technology, which has two operating modes: series and parallel.

#### 5.3.3. C. Passive and Active Energy Harvesting

**Figure 17:**
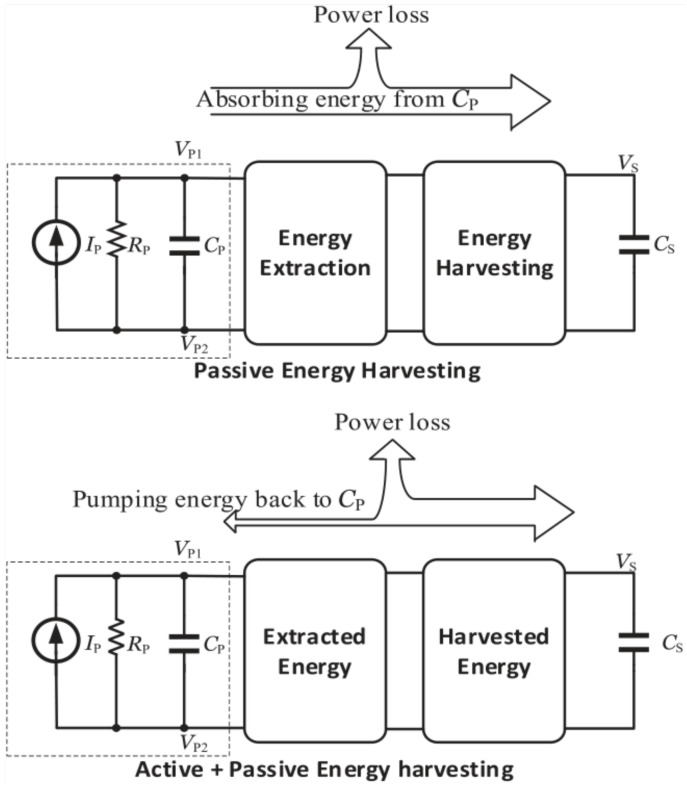
Hybrid active and passive energy harvesting [62].

The majority of energy harvesting techniques rely on gathering energy from an external passive component (such as an inductor or capacitor), which is subsequently utilized to charge and discharge $C_{P}$ in order to maximize energy harvesting potential. Active energy harvesting is a different process that was initially presented in [59]. Combining hybrid active and passive schemes, investing a tiny amount of extracted energy for PEH excitation, and then recovering a much greater harvested energy have been the driving forces behind some recent research (Figure 16). Following energy injection or a prebiasing process, the voltage change may be more pronounced [28,60,61]. More energy can be gathered in return when the flipping voltage amplitude is increased by a little amount of pumping energy back to the PEH’s intrinsic capacitance. Liang et al. combined several passive voltage flipping motions with an active energy injection step in [51]. This was made possible by a bias capacitor’s self-adaptive bias voltage. As an active component that may self-charge and discharge, the bias capacitor ensures that the vibration level can adjust. Zhao et al. recently presented a bidirectional interface circuit in [62] to allow for self-exciting PEH, Figure 17. The use of both active and passive energy harvesting strategies resulted in a significant improvement in energy extraction performance. In this design, the storage capacitor has two purposes: Firstly, it stores the extracted energy; secondly, it supplies bias voltage for the two bias-flipping phases that are carried out using the synchronized multiple bias-flip (SMBF) approach. Stated differently, the storage capacitor functions as an active element to continuously vibrate and charge $C_{P}$ during stimulation.

In the final technical section, hybrid approaches are presented as a promising avenue to merge the advantages of multiple energy harvesting methods. The summary reflects on how combining capacitor–inductor circuits with traditional SSHI and MPPT techniques can yield more compact, efficient, and adaptable systems, setting a forward-looking agenda for integrating these innovations into practical, real-world applications.

## 6. Discussion of Performance Enhancement

**Figure 18:**
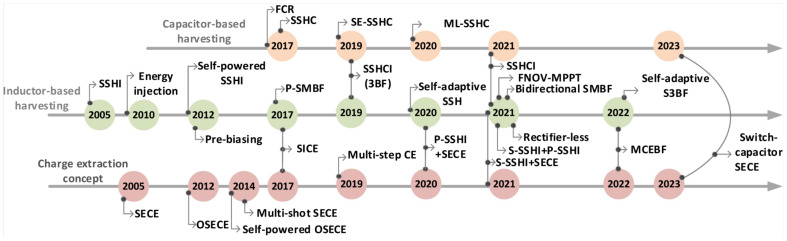
Progress on the synchronous switch harvesting energy over the years of 2005–2023 [65].

Table 1 and Table 2 quantitatively assess the performance enhancements achieved by the interface circuits under review, utilizing the Maximum Output Power Improvement Rate (MOPIR) and conversion efficiency (η) as key performance indicators. These technologies are summarized and visually represented in Figure 18, which illustrates the progress made over the years 2005–2023.

(5) $$\mathrm{MOPIR}=\frac{\max(P_{\mathrm{out}-\mathrm{TECH}})}{\max(P_{\mathrm{out}-\mathrm{FBR}})}\times 100$$

(6) $$\eta =\frac{P_{\mathrm{out}}}{P_{\mathrm{out}}+P_{\mathrm{loss}}}\times 100$$

These metrics, rigorously defined and widely adopted within the power electronics and energy harvesting research communities, as evidenced by publications in prestigious journals such as *IEEE Transactions on Power Electronics*, *IEEE Journal of Solid-State Circuits*, *IEEE Transactions on Industrial Electronics*, and *IEEE Transactions on Circuits and Systems I and II*, provide a standardized framework for objective comparison. Specifically, MOPIR, formulated as in Equation (5) and defined by Xie et al. [63], normalizes the power improvement relative to the conventional full-bridge rectifier (FBR), while the conversion efficiency η, detailed in Equation (6) and defined by Badr et al. [64], quantifies the effectiveness of power conversion by considering power losses.

**Table 1:** Comparison of Interface Circuits for Piezoelectric Energy Harvesting.

Interface	Technology	Voc (V)	Power (µW) and Efficiency	Frequency (Hz)	Key Features/PZT	Load Depend./SP
FTSCR [63]	0.18 µm CMOS	3	4.84 µW, 8.14x	57	C = 800 pF, On-Chip, PZT5A	No, No
SICE [53]	Discrete	5.2	380 µW, 5x	N/A	L1 = L2 = 1 H, Custom PZT	No, No
FCR [66]	0.18 µm CMOS	2–8.5	50.2 µW, 4.83x	110 k	C = 1.44 nF On-Chip, MIDE V21B, V22B	Yes, No
SE-SSHC [2]	0.18 µm CMOS	2.5	186 µW, 8.2x	219	Ctotal = 4 nF, On-Chip, Custom MEMS	Yes, No
MSBF [67]	0.13 µm CMOS	1.5	63 µW, 4.48x	441	L = 47 µH, Cb = 14 nF, Cross-coupled FBR, Off-Chip, MIDE PPA-1022	Yes, No
S-SSHI-phi [6]	0.25 µm HV CMOS	2.5	228.8 µW, 8.27x	126.7	C = 15.9 nF, Cross-coupled FBR, Mide V22B	Yes, No
FCR Split-Phase [5]	0.18 µm CMOS	3	33.1 µW, 9.2x	200	C = 22 nF, Cross-coupled P/NMOS/Frac Voc, Mide PPA-1021	Yes, No
Sense/set MPPT [39]	0.18 µm CMOS	1.5	2.76 µW, 5.41x	85	C = 10 nF, MOS switches/Frac Voc, Mide PA-1022	Yes, No
SECE tuning/sensing [8]	0.6 µm CMOS	3	275 µW, 3.31x	56	C = 24 nF, Cross-coupled FBR P&O, Frac Voc	Yes, No
VM-SECE [10]	0.35 µm CMOS	4	13.5 µW, 2.43	90–160	C = 17–49 nF, Active FBR	Yes, No

Abbreviations: CMOS: complementary metal-oxide semiconductor; BiCMOS: bipolar CMOS; MPPT: maximum power point tracking; PZT: lead zirconate titanate; SP: self-powered; SSHI: synchronized switch harvesting on inductor; SECE: synchronous electric charge extraction; FBR: full-bridge rectifier; SEC: switched electrical circuit; efficiency = P_out_/P_out(FBR)_.

**Table 2:** Comparison of Interface Circuits for Self-Powered Systems.

Interface	Technology	Voc (V)	Power and Efficiency	Frequency (Hz)	Key Features/PZT	Load Depend./Year
P-S3BF [51]	Discrete	15	470 µW, 2.87x	24.9	L = 47 µH, Cb = 4.7 µF, Off-Chip, PZT-5A	Yes, 2018
SSHCI [48]	0.18 µm CMOS	3.2	13 µW, 5.44x	415	L = 100 µH, C = 2 nF, Off-Chip, Custom MEMS	Yes, 2021
ML-SSHC [68]	0.18 µm CMOS	1.27–2.34	1.51–4.85 µW, 8.7x	22	Ctotal = 600 pF, On-Chip, VH1504C-2	Yes, 2020
SSHC [34]	0.35 µm CMOS	2.5	161.8 µW, 9.7x	92	C = 45 nF, Off-Chip, MIDE V21BL	Yes, 2017
S-SSHI+SECE [52]	Discrete	6	280 µW, 7.28x	18	L1 = L2 = 2.21 mH, MIDE PPA-2011	Medium, 2020
P-SSHI+SECE [52]	Discrete	3	85.7 µW, 2x	23	L = 1.5 mH, PZT-5H	Medium, 2020
S/P-SSHI [54]	Discrete	5	112 µW, 3.2x	70	L = 1.5 mH, Custom PZT	Medium, 2021
NYC-PSSHI [69]	Discrete	3–7	Up to 300 µW, 8.21x	100–500	L = 100 µH, MIDE PPA-1001	Yes, 2018
P-SSHI [20]	-	1.8	11.15 µW, -	5	optimal load: Ropt = 400 kΩ	Yes, 2017
Adapt. SSHI [45]	0.18 µm CMOS	-	11.15 µW, 4.6x	5	optimal load: Ropt = 400 kΩ	Yes, 2020
ReL-SSHI [46]	0.18 µm CMOS	1.6–1.8	-, 4.6x	18.6	-	-, 2021
ZSECE [18]	Discrete	-	-, 7.93x	18.6	-	No, 2022
BL-S-SSHI [19]	Discrete	-	-, 3x	18.6	voltage doubler	-, 2021
MI-P-SSHI [17]	-	-	-, -	-	phase mismatch of multiple sources	-, 2022
SPEDS-SSHI [16]	-	5.2–5.8	-, 4.6x	-	RC differential circuit, extensible	Yes, 2022
CHP-AM [70]	Discrete	16.5	170 µW, 1.89x	24	circular hole-based	Yes, 2024
SP-MIHR [71]	-	4.5–6.5	-, 4.7x	-	multiple PZTs, arbitrary phase difference, low start-up voltage,	Yes, 2024
MSP-SICE [72]	Discrete	1.88–6.5	215 µW, x	8–8.4	$L_{1}=L_{2}=10$ mH, PZT-4	Yes $R_{opt}=200$ kΩ, 2024
SS-TEPG [73]	-	30.5–151	1.9–8.2 mW, x	8.3–31.2	LTC 3588-based hybridized power management (L-HPM)	Yes, 2024
AR-SBC [74]	0.18 µm HV	3.9	14.1 mW @ 1 kΩ, x	20	self-biased comparator	Yes $R_{opt}=50$ kΩ, 2024
EP–SSHI–TEA [75]	Discrete	6–8	400 µW, 3.63x	34	multiple PZTs	Yes, 2023
SP-P-PV [76]	180 nm CMOS	3.3/0.6	189 µW, -	43	L1 = L2 = 2.21 mH, C1 = C2 = 220 µF, Vsto = 22 µF, PZT-5H	Yes, 2024
A-pre-bias-MPPT [77]	0.18 µm HV	0.3–1	32.44 nW, 12.68x	146	L = 47 µH,SADPM	Yes, 2024
GI-DR [78]	-	15	3.17 µW, 7.63x	20	$C_{sto}=1000$ mF, $L_{s}=150$ mH, $R_{sn}=250$ Ω, gyrator-induced,	Yes, 2024
HTG [79]	-	4.5–6.5	14.58 mW, 4.7x	8	$R_{sn}=20$ MΩ	Yes, 2024

Abbreviations: CMOS: complementary metal-oxide semiconductor; BiCMOS: bipolar CMOS; MPPT: maximum power point tracking; PZT: lead zirconate titanate; SP: self-powered; SSHI: synchronized switch harvesting on inductor; SECE: synchronous electric charge extraction; FBR: full-bridge rectifier; SEC: switched electrical circuit; efficiency = P_out_/P_out(FBR)_.

Our comparative analysis definitively demonstrates that while traditional rectifier-based circuits, such as the FBR and half-bridge rectifier, offer design simplicity, their achievable performance is fundamentally constrained by inherent limitations. These limitations primarily stem from voltage drops across rectifying diodes and suboptimal impedance matching to the piezoelectric transducer, thereby restricting the overall power extraction efficiency. Conversely, a significant paradigm shift in performance is observed with advanced interface circuits employing synchronous switching techniques. Methodologies like Synchronous Electric Charge Extraction (SECE) [2] and Synchronous Switch Harvesting on Inductor (SSHI) schemes [34] effectively overcome these limitations, enabling substantially higher power extraction by actively managing the charge transfer and optimizing impedance conditions. Inductor-based hybrid interface circuits, as explored in studies by Xia et al. [52,54,80], further exemplify this trend. Their ease of implementation with minimal discrete components belies their capacity to achieve high MOPIR, particularly under conditions of higher input excitation, showcasing the benefits of inductive elements in resonant energy harvesting scenarios. Furthermore, leveraging CMOS technology for techniques like multiple bias flipping, as investigated by Javvaji et al. [67], has been shown to yield MOPIR enhancements exceeding fourfold, demonstrating the effectiveness of integrated circuit implementations for advanced control. As highlighted by Badr et al. [64], achieving high output power levels enables the implementation of self-powered, precise switching control mechanisms, such as in P-SSHI, which in turn minimizes power dissipation associated with control overhead.

**Figure 19:**
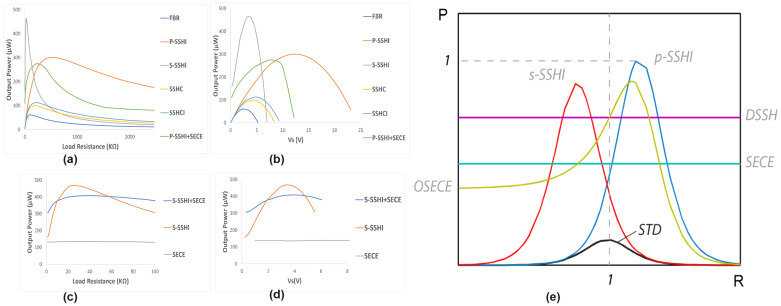
Comparison results of various interface circuits in the case study. (**a**) Output power versus resistance load. (**b**) Output power with respect to rectified voltage. (**c**) Output power versus resistive load with and without hybrid method. (**d**) Output power versus rectified voltage with and without hybrid method. (**e**) Comparison of harvested power for the references earlier topologies.

To further clarify these performance characteristics, LTspice simulations were performed using a representative piezoelectric energy harvester (PEH) model. The simulations evaluated the various interface circuits listed in Table 1, and the top-performing configurations are summarized in Table 3. As illustrated in Figure 19a, these simulations underscore the critical role of Maximum Power Point Tracking (MPPT) modules, especially for SSHI-based techniques. The inherent load dependency of SSHI circuits necessitates MPPT for optimal impedance matching and maximized power extraction. The reduced power observed in SSHCI [48] simulations, as seen in Figure 19a, can be attributed to the increased complexity of its digital control unit required for managing more intricate switching phases, highlighting a trade-off between control sophistication and overall power efficiency. While SSHC configurations which eliminate the bulky inductor achieve inductor size reduction, this comes at the cost of lower output power due to capacitor-based configuration limitations and more complex switching requirements. Figure 19b further clarifies that the optimal rectified voltage ($V_{s}$) in S-SSHI is inherently lower than that in P-SSHI configurations, a key design consideration for system integration. As anticipated from theoretical analysis, SECE demonstrates a near-linear output power response across load variations, indicating its inherent load independence, whereas hybrid techniques combining S-SSHI and SECE, as shown by Xia et al. [52] [Figure 19c], effectively smooth out the power output profile of S-SSHI, enhancing its robustness under varying load conditions. Furthermore, as depicted in Figure 19d, varying the rectified voltage reveals the output power of SECE to be largely insensitive to voltage changes, contrasting with S-SSHI whose output power is significantly affected with the hybrid technique exhibiting a reduced sensitivity compared to standalone S-SSHI.

The Self-Powered Multi-Input Hybrid Rectifier (SP-MIHR) [71] stands out as a particularly advanced solution, showcasing a superior end-to-end conversion efficiency of 67.3% and a maximum output power 1.47 times greater than classical S-SSHI circuits. Notably, the SP-MIHR also achieves a 0.5 V reduction in start-up voltage and exhibits a 4.12-fold power improvement and 1.3-fold voltage enhancement over standard multi-input energy harvesting circuits, all while maintaining a structurally simple and scalable architecture [4]. While SSHC [34] offers a compromise between complexity and implementation ease by using off-chip components and SSHCI [48] reduces inductor size using a capacitor-inductor hybrid approach (albeit still with off-chip components), SE-SSHC [2] and FTSCR [63] represent alternative topologies prioritizing ease of implementation by eliminating the need for off-chip components altogether.

In addition to interface circuit innovations, advancements in transducer designs also play a critical synergistic role in enhancing overall energy harvesting system performance. For example, the circular hole-based piezoelectric energy harvester demonstrates a substantial 13.8% increase in open-circuit voltage, resulting in an approximate 89% improvement in harvested energy. The practical efficacy of these combined advancements is further underscored by the successful integration of such harvesters with low-power voltage regulators [2], highlighting their suitability for real-world self-powered system implementations.

However, despite the significant performance gains achieved in laboratory settings, the scalability of these advanced interface circuits for industrial-scale piezoelectric energy harvesting remains a crucial consideration for widespread practical deployment. Maintaining the demonstrated high efficiency and performance metrics as production scales presents substantial challenges. These include mitigating the impact of component variations inherent in mass manufacturing, managing increased parasitic effects in larger circuit layouts, and ensuring the robust and consistent operation of complex control strategies in mass-produced circuits. Furthermore, the economic viability of implementing sophisticated control circuitry, particularly for techniques like SECE and SSH, needs careful evaluation against the cost-effectiveness and robustness of simpler circuit designs for different application contexts. Addressing these scalability challenges requires focused future research and development efforts aimed at optimizing designs for manufacturability, developing robust and easily implementable control strategies, and carefully selecting cost-effective components without compromising performance. Such efforts are essential to bridge the gap between laboratory-proven performance and the practical feasibility of deploying high-performance interface circuits in large-scale piezoelectric energy harvesting systems.

In conclusion, realizing truly optimal piezoelectric energy harvesting necessitates a careful balancing of inherent trade-offs [1]. Key design considerations include circuit complexity, the precision and adaptability of calibration methodologies [9], and the effectiveness of impedance matching strategies, including those achieved through intentional delays in switching timing as discussed in state-of-the-art reviews [81]. These factors become particularly critical in complex systems integrating multiple piezoelectric transducers, especially those operating under diverse and asynchronous vibrational excitations. A judicious balance across these design parameters is paramount to fully unlock the potential of advanced energy harvesting interface circuits and facilitate their widespread adoption in practical self-powered applications.

## 7. Conclusions

The evolution of interface circuits for piezoelectric energy harvesting in self-powered systems is propelled by a continuous drive for enhanced efficiency, miniaturization, and intelligent control. This relentless pursuit is reflected in current trends focusing on advanced rectification techniques, adaptive impedance matching algorithms like Maximum Power Point Tracking (MPPT), and sophisticated energy management systems—all aimed at maximizing power extraction and storage. As highlighted in this comprehensive review of power conditioning techniques, these advancements mark a significant stride from traditional rectification towards cutting-edge strategies. Our investigation reveals that innovative circuit topologies like the SP-MIHR, SPEDS-SSHI, ZSECE, MI-P-SSHI, BL-S-SSHI and P-SSHI alongside transducer advancements such as the circular hole-based harvester are key enablers for enhanced efficiency, increased power output, and improved robustness in self-powered piezoelectric energy harvesting systems. Key findings underscore the superior performance of advanced topologies utilizing synchronous switching and dynamic impedance matching and further emphasize the multifaceted benefits of the mentioned topologies, while innovative transducer designs demonstrably amplify system performance. Further progression in this field includes System-on-Chip (SoC) integration for footprint reduction, low-power design for minimized losses, and application-specific optimizations tailored for diverse applications ranging from wearables and IoT devices to structural health monitoring. While both piezoelectric and magnetoelectric (ME) devices are employed for energy harvesting in self-powered systems, their differing transduction mechanisms necessitate distinct interface circuit designs. Piezoelectric harvesters, converting mechanical strain to electrical energy, typically generate high-voltage, low-current AC signals, optimally paired with high-impedance circuits. In contrast, magnetoelectric harvesters, which harness magnetic field variations, often produce lower voltage and current levels with lower impedance, sometimes even DC output. Consequently, directly applying a piezoelectric-specific interface circuit to an ME harvester would likely result in significant power loss and inefficient operation due to impedance mismatch and incompatible voltage/current handling. Adapting interface circuits to match the specific output characteristics, frequency response, and potential biasing needs of ME devices is therefore crucial for achieving optimal energy harvesting efficiency in self-powered systems utilizing magnetoelectric conversion. These collective advancements, including circuits tailored for specific transducer types, pave the way for viable self-powered systems. However, realizing widespread adoption requires strategically tackling persistent challenges, notably in control circuit power consumption, scalability, long-term reliability, and environmental impact. This review provides a comprehensive overview of recent advancements in interface circuits for piezoelectric energy harvesting systems, highlighting innovative designs, improved control methodologies, and enhanced power extraction techniques. Notably, the continuous development of advanced methods such as SECE, SSH, and active MPPT algorithms are addressing long-standing challenges in efficiency and reliability. However, as we discuss, scalability remains a significant hurdle for industrial-scale implementation. In addition, the integration of intelligent control strategies and the exploration of eco-friendly materials are promising directions for future research. Addressing these areas is critical to advancing the practical deployment of self-powered systems across applications ranging from wearable electronics to structural health monitoring. Future research must therefore prioritize broadening comparative analyses, optimizing low-power control, rigorous real-world validation, and addressing sustainability through life cycle assessments and eco-friendly materials. Ultimately, by overcoming these hurdles and continuing on the current trajectory of development, the field of piezoelectric energy harvesting, alongside adapted approaches for magnetoelectric systems, is poised to deliver highly efficient, robust, and adaptable interface circuits. These circuits will be crucial enablers for the widespread deployment of truly self-powered systems across diverse fields, fostering a more energy-independent and environmentally conscious future.

## Figures and Tables

**Table 3:** Comparison of top Different Circuits.

Circuit Name	Advantages	Limitations/Criticisms	Rating/Effectiveness and Complexity
SPEDS-SSHI [16]	Simultaneous energy harvesting from multiple PZTs, Self-powered operation, Improved harvested power and efficiency, Reduced number of switches and enhanced component reuse, Ability to harvest energy from PZTs with any phase difference, Enhanced system reliability and stability	Lack of experimental validation, Limited scope of comparison, Lack of detailed performance metrics, Practical implementation challenges	4/5
Multi-input P-SSHI Interface Circuit [17]	Efficient energy extraction from multiple input sources, Improved rectifier circuit topology, Self-powered strategy for enhanced efficiency, Simplified structure with reduced switches and inductors, Unique characteristics in the output voltage waveform, Cost and feasibility	Lack of empirical validation, Scalability and robustness, Limited comparison with existing approaches, Environmental considerations	3.5/5
Self-powered Extensible SECE Rectifier [18]	Enhances power output for piezoelectric energy harvesting, Enables battery-free embedded devices, Provides load-independent power output, Efficient energy extraction from piezoelectric transducers, Simplifies system design and reduces costs	Lack of experimental validation, Limited scope of application, Complexity and cost of implementation, Trade-offs and efficiency compared to alternatives	4/5
Self-powered Multi-Input Bridgeless series SSHI Circuit [19]	Integrates voltage doubler topology, Maximizes power available for the load, Simplifies circuit complexity, Achieves threefold increase in power harvesting, Autonomous operation without external power supply, Potential applications in wireless sensor networks and more	Limited scope of the research, Lack of comprehensive comparison, Limited experimental details, Lack of real-world validation, Scalability and adaptability concerns, Reproducibility and generalizability issues	3.5/5
Optimized Self-Powered P-SSHI Circuit [20]	Enhances efficiency and reliability of energy harvesting converters, Utilizes bipolar transistors for increased power output, Replaces energy-consuming components with more efficient alternatives	Lack of detailed information, Limited scope, Incomplete explanation, Methodological concerns, Lack of external validation	3/5

## Data Availability

Not applicable.
